# Seeking Solvation: Exploring the Role of Protein Hydration in Silk Gelation

**DOI:** 10.3390/molecules27020551

**Published:** 2022-01-16

**Authors:** Peter R. Laity, Chris Holland

**Affiliations:** Department of Materials Science and Engineering, University of Sheffield, Sir Robert Hadfield Building, Mappin St., Sheffield S1 3JD, UK

**Keywords:** fibroin, native silk feedstock, protein hydration shell, gelation, thermodynamic model

## Abstract

The mechanism by which arthropods (e.g., spiders and many insects) can produce silk fibres from an aqueous protein (fibroin) solution has remained elusive, despite much scientific investigation. In this work, we used several techniques to explore the role of a hydration shell bound to the fibroin in native silk feedstock (NSF) from *Bombyx mori* silkworms. Small angle X-ray and dynamic light scattering (SAXS and DLS) revealed a coil size (radius of gyration or hydrodynamic radius) around 12 nm, providing considerable scope for hydration. Aggregation in dilute aqueous solution was observed above 65 °C, matching the gelation temperature of more concentrated solutions and suggesting that the strength of interaction with the solvent (i.e., water) was the dominant factor. Infrared (IR) spectroscopy indicated decreasing hydration as the temperature was raised, with similar changes in hydration following gelation by freezing or heating. It was found that the solubility of fibroin in water or aqueous salt solutions could be described well by a relatively simple thermodynamic model for the stability of the protein hydration shell, which suggests that the affected water is enthalpically favoured but entropically penalised, due to its reduced (vibrational or translational) dynamics. Moreover, while the majority of this investigation used fibroin from *B. mori*, comparisons with published work on silk proteins from other silkworms and spiders, globular proteins and peptide model systems suggest that our findings may be of much wider significance.

## 1. Introduction

The enigmatic ability of some arthropods (notably spiders and many insects [1,2,3,4,5,6,7,8]) to spin silk fibres from an aqueous protein solution (feedstock) under ambient conditions has attracted much scientific interest. Yet, key questions remain concerning the physical state of the protein in the feedstock and the mechanism by which it solidifies to the silk fibre. Many previous authors have attempted to address these questions, although a complete understanding remains elusive. Often, the explanations offered to date are not entirely satisfactory, in that they do not fully encompass (or may even be contradicted by) other experimental observations.

In previous work with native silk feedstock (NSF) from *Bombyx mori* silkworms, we suggested the importance of a hydration shell on the fibroin, which stabilises the protein in a solution [9]. This can be displaced under certain circumstances, allowing hydrogen bonds (H-bonds) to form between peptide groups, leading to gelation. We proposed that this hydration shell is thermodynamically stable (by up to about 150 J mol^−1^ of water), through favourable H-bonding with the peptide groups, thereby protecting the silkworm from premature gelation. The water molecules constituting this shell are entropically penalised (by around −2.2 J mol^−1^ K^−1^, relative to free water); however, since their molecular motion (rotation and translation) were reduced by being ‘enslaved’ to the protein. Remarkably, this hypothesis can explain the various apparently disparate ways that silk gelation can occur.

- By raising the temperature (*T*) [9,10,11,12,13,14,15,16], the entropic component (*T*·Δ*S*) becomes more important, eventually dominating the enthalpy (Δ*H*) and rendering the hydration shell thermodynamically unstable above about 65 °C.
- The addition of other water-soluble species (e.g., salts or alcohols [17,18,19]) reduces the free energy of the bulk aqueous phase below that of the hydration shell, ‘enticing’ water molecules away from the protein.
- Similarly, freezing the protein solution [9,16,20,21,22] produces a lower energy state for the water (i.e., as ice), thereby enticing it to leave the hydration shell. This example is particularly instructive, as it is the increased entropy of liquid water overcoming the stronger enthalpic component of ice crystals that defines its melting point.
- Most importantly, from the perspective of natural silk spinning, flow stress [8,9,10,20,23,24,25,26,27,28,29,30,31] causes the fibroin chain to deform away from an equilibrium shape. Under these conditions, the hydration shell becomes unstable through a further loss of entropy, or due to some peptide groups being forced to adopt conformations incompatible with the amount of H-bonding required to maintain stability.

Sceptics may claim that these ideas are somewhat fictive, conflicting with what is often assumed regarding protein solutions. The conventional view is that soluble proteins adopt precisely folded globular structures, with hydrophilic amino acids towards the exterior (in contact with water), while hydrophobic amino acids are buried within the core (away from bulk water) [32,33,34,35,36,37,38,39]. According to this view, denaturation involves ‘melting’ of the coil structure, with aggregation following from unfavourable interactions between water and the exposed hydrophobic amino acids.

By contrast, our hypothesis depends on understanding fibroin in NSF to be a ‘random-coil’ polymer, undergoing uniform molecular motion under roughly ‘theta’ conditions. In this respect, the native fibroin appears structurally similar to recently characterised intrinsically disordered proteins (IDP), which perform various important physiological roles in spite of not having fixed tertiary structure [40,41,42,43,44]. Moreover, this view of fibroin is well supported in the literature. Over 50 years ago, using optical rotatory dispersion (ORD) and circular dichroism (CD), Iizuka [45] demonstrated that *B. mori* fibroin in dilute aqueous solutions (prepared directly from NSF or after dissolving fibre using concentrated LiBr solution and dialysing) presents a disordered conformation. Based on various nuclear magnetic resonance (NMR) methods, Asakura and co-workers [28,46,47,48] suggested that ‘the highly concentrated silk solution contained in the middle silk gland has residues in energetically favored conformations close to average random coil values, but forms a hydrogen-bonded network that keeps it in a repeated type II β-turn structure’. Other studies have also demonstrated predominantly random coil, helical or β-turn conformations in various native, recombinant or redissolved spidroin (spider silk protein) solutions [49,50,51,52,53,54,55,56,57].

In evaluating these various published results, it should be noted that several NMR methods [58] and CD [59] are sensitive to the secondary structure of the protein (i.e., orientation of adjacent amino acids), whereas small-angle scattering (SAS) provides more course-grained indications of tertiary structure (i.e., overall molecular shape). Hence, in the recent study of spidroins by Greving et al. [57], CD suggests there may be limited flexibility between some adjacent amino acids, leading to short-range structure, while greater flexibility over longer segments allows the proteins to approximate to random coils in SAS methods.

It may also be noted that fibroins appear to be exquisitely sensitive to mechanical or chemical stimuli. In addition to gelation due to flow stress [8,9,10,20,23,24,25,26,27,28,29,30,31], the β-sheet component may increase due to the natural passage of fibroin along the silk gland and associated changes in pH or ionic environment [50,51,52,53]. Moreover, conformational changes may arise as artefacts of inappropriate sample preparation methods (e.g., involving salts, freezing or excessive stress during handling); thus, a certain amount of circumspection is advisable when considering some of the claims reported in the literature.

Further support for our view can be found from small-angle neutron scattering (SANS) data published by Greving et al. [60], for diluted fibroin sampled directly from *B. mori* silk glands. Using Guinier analysis, it appeared that the radius of gyration (*R_G_*) of fibroin ranged from 8 to 16 nm, with larger values at lower concentrations. Taking an average from their data (omitting the lowest concentration solution, which presented considerable uncertainty), the average value of *R_G_* appeared to be around 11 to 12 nm.

Although those authors did not report it themselves, their data (from 2.5 to 37.6 mg mL^−1^) can be fitted well using the Debye model [61], which provides a good approximation for scattering from a ‘Gaussian’ chain adopting a ‘3-dimensional random walk’ conformation:

(1a) $$D\left(q\right)=\;\frac{2I_{0}\cdot \left(e^{-x}+x-1\right)}{x^{2}}$$

where:

(1b) $$x=\;\frac{q^{2}Nb^{2}}{6}\;=\;q^{2}R_{G}^{2}$$

*I*_0_ is a scaling factor that represents the overall strength of the scattering, sample volume, acquisition time, intensity of illumination and the detector response, while *q* is the scattering vector:

(1c) $$q\;=\;\frac{4\pi \cdot sin\theta }{\lambda }$$

where *2θ* is the scattering angle and *λ* is the wavelength. Fitting this model to the published scattering curves yielded values for *R_G_* around 11 to 12 nm, which agreed with the values reported by Greving et al. based on their Guinier analysis. Moreover, that also concurs with a predicted value of 12.1 nm, given by [62]:

(2) $$R_{G}=\;\sqrt{\frac{Nb^{2}}{6}}$$

for a ‘freely jointed’ chain under ‘theta solution’ conditions, consisting of *N* = 5525 amino acids (i.e., based on the published sequences for the conjoined ‘heavy’ Fib-H and ‘light’ Fib-L chains of *B. mori* [63]), with a monomer length (*b*) of 0.4 nm [64].

As all the solutions studied by Greving et al. were dilute (below 38 mg mL^−1^, i.e., concentrations too low for chain overlap), we speculate that apparent variations in *R_G_* may have been due to the Ca^2+^ concentrations in the solutions. It is known that Ca^2+^ in the NSF can form ionic crosslinks (calcium bridges) between carboxylate-substituted amino acids, thereby raising the viscosity through ‘sticky reptation’ [65,66,67]. It is possible that a similar mechanism in dilute solutions could resist coil expansion (i.e., giving smaller *R_G_* values).

Smaller values of *R_G_* have been reported for redissolved (regenerated) *B. mori* cocoon silk. Martel and coworkers reported a slightly lower value (10.8 nm) [68], while Greving et al. [60] reported considerably lower values (<5.5 nm). It seems likely, however, that these differences can be explained by depolymerisation ding the degumming and redissolution processes.

The published sequence gives a molecular weight of around 419.6 kDa for the complete Fib-H-Fib-L chain. Hence, to put this in context, a *R_G_* of 12 nm corresponds to a density of around 0.045 g cm^−3^ for amino acids within each protein coil in the diluted NSF. Clearly, this suggests considerable scope for water to be incorporated within the coil; thus, the highly polar peptide groups (dipole moment = 3.45 D [69,70]) in the backbone are likely to be extensively hydrated. Even at the much higher protein concentration in undiluted NSF (ca. 0.25 g cm^−3^ [65]), the water to peptide ratio is around 12.6:1 (using the average amino acid formula weight of 75.9 g mol^−1^, based on the published sequences [63]). In view of the uniform dynamics revealed by NMR of intact silkworms [47,48], this implies considerable hydration of the protein backbone in NSF.

Several relations have been proposed between the chain length (i.e., the number of amino acids) and radius of gyration for proteins [71,72,73,74,75]. These predict *R_G_* values around 4.5 nm, for *B. mori* fibroin as a natively folded globular protein, 16 to 23 nm as a chemically denatured globular protein (e.g., using urea or guanidine hydrochloride), or 28 to 32 nm as an IDP. Thus, the fibroin coil appears to be considerably more expanded than a typical globular protein, suggesting a greater level of hydration; yet the effect is not as great as that achieved for globular proteins in aqueous urea or guanidinium hydrochloride solutions, or typical IDPs.

Several factors may contribute towards the smaller *R_G_* of the native fibroin chain compared with chemically denatured proteins or IDPs. Firstly, as noted above, there may be calcium bridges between carboxylate-containing amino acids in fibroin, which resist chain expansion. Secondly, the greater expansion of IDPs may be due to their compositional bias towards higher concentrations of polar (Asn, Gln) and charged (acidic: Asp, Glu; basic: Lys, Arg) amino acids [43,76]. For example, Lys (an essential amino acid, fairly abundant in mulberry leaves [77,78]) constitutes around 40% of the amino acids in some IDP sequences [79], while fibroin contains a considerably smaller amount of alcohol substituted amino acids (12% Ser and 1% Thr) but relatively few polar (1.2%) or charged amino acids (1.4% acidic, 0.9% basic) [63]. Note: *B. mori* fibroin also contains a fairly large (around 5.5%) amount of Tyr, which suggests it may have an important function; however, the phenolic side group is expected to be considerably less hydrophilic than Ser or Thr. Polar and charged side groups are likely to attract additional hydration. In addition, charged amino acids (and their counter-ions) may also exhibit electrostatic repulsion. Hence, both effects could contribute to the increased expansion of IDPs.

There may also be questions regarding structure within or interactions between the terminal segments of fibroin. Several authors have suggested that the terminal segments undergo pH-dependent aggregation, which plays an important part in spinning silkworm [80] and spider silks [81,82,83,84,85,86,87,88,89]. This can be ruled out for the NSF solutions studied by Greving et al. however, which were prepared using deionised water, so that the neutral pH (ca. 7) is not expected to induce aggregation. As further support for this, the molecular weights obtained from the scattering data were consistent with single fibroin molecules. Indeed, it may be suggested that the higher abundance of ionisable amino acids may render the terminal segments more like IDPs under these pH conditions, exhibiting greater swelling compared with the more repetitive main segments of the fibroin chain, although the net effect was insufficient to be revealed by the SANS data.

This picture of a hydrated fibroin coil in NSF appears to conflict with suggestions from other authors that the protein is dominated by segments of hydrophobic amino acids [12,14,90,91,92,93,94,95], based on the Kyte and Doolittle hydropathy index [96]. We suggest this is a misinterpretation of Kyte and Doolittle’s original (1982) paper, however, which considered the likelihood of finding the various amino acids in the core or at the surface of globular proteins. While it is likely that hydrophobic amino acids would be accommodated within the core, rather than being exposed at the hydrated surface, it is also likely that the smaller amino acids (Gly and Ala) could be incorporated into the core, as their side groups present less stereochemical restrictions to packing amongst the larger amino acids. Thus, we suggest a more reliable picture emerges from more recent (1993) work by Privalov and Makhatadze [97,98], which found that the main amino acid units in fibroin (Gly, Ala and Ser) are strongly hydrophilic, largely due to the H-bonding capabilities of the peptide-linked backbone.

To summarise, we suggest the evidence points to fibroin in NSF (from *B. mori* and other animals) being largely hydrophilic, with characteristics (i.e., approximating to random coil geometry and uniform dynamics) typical of a water-soluble polymer in solution [46,47,48,49,99]. Clearly, this precludes a number of suggestions for how the phase change to a solid fibre occurs (e.g., through hydrophobic interactions or further interactions between liquid crystalline aggregates pre-existing in the NSF). From this starting point, we examined our ‘hydration shell’ hypothesis [9] in greater detail in the present work, using a combination of different approaches.

- Firstly, the molecular size of *B. mori* fibroin was investigated in dilute solution, using small-angle X-ray scattering (SAXS) and dynamic light scattering (DLS), at room temperature and during heating to 80 °C.
- Infrared (IR) spectroscopy was used to probe thermally driven changes in the hydration of NSF and several amide-containing model compounds.
- Changes in hydration associated with gelation of NSF by freezing and thawing were also investigated using IR spectroscopy.
- Turbidity and protein aggregation measurements were used to investigate the solubility of diluted fibroin in salt solutions, where the free energy of the bulk aqueous phase can be determined.

We demonstrate that these results can be explained remarkably well by our hypothesis, based on restricted dynamics of water within the hydration shell, which is discussed in the broader contexts of protein and polymer science.

## 2. Results

### 2.1. Protein Coil Size by SAXS

**Figure 1:**
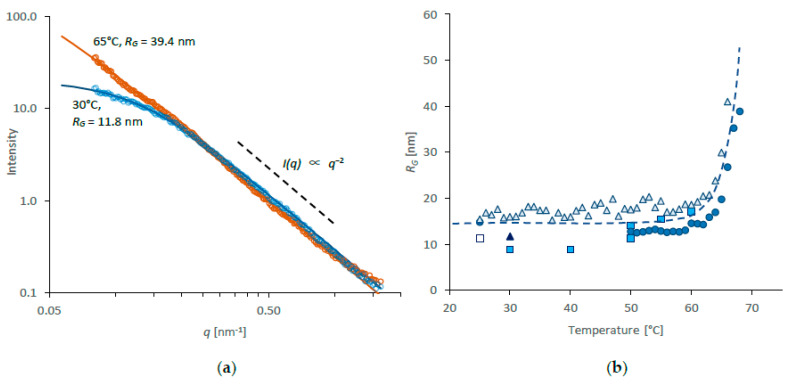
SAXS data for diluted NSF specimens: (**a**) individual markers represent intensity data (on logarithmic axes, to show the most important features) measured at 30 (blue)and 65 °C (brown); the continuous lines represent the Debye model (Equation (1)) with the values of *R_G_* shown; the dashed line shows the slope expected for ‘power law’ scattering from a polymer in solution; (**b**) values of *R_G_* obtained for diluted NSF during heating; the different shapes and colours represent different specimens; the dashed line merely provides a guide for the eye.

SAXS data for NSF taken directly from MP silk glands and diluted in water (to around 1.2% *w*/*w* of protein) are shown in Figure 1a, on logarithmic axes to reveal the most important features. As commonly found for scattering in the small-angle range, the intensity decreased along a smooth curve towards larger angles (larger q). Nevertheless, several important details could be inferred from the data.

Notwithstanding the different scattering mechanisms of X-rays and neutrons (the former interact with electrons through the electromagnetic force, while the latter interact with nuclei through nuclear forces [61]), the SAXS data appeared essentially similar to the SANS data for diluted NSF reported previously by Greving et al. [60]. In both cases, the plots (on logarithmic axes) became significantly steeper beyond 0.16 nm^−^^1^ (0.016 Å^−^^1^) and almost straight, with the scattered intensity following a *q^−^*^2^ power law dependence. This is commonly observed for polymer chains in solution, which may be regarded as ‘fractal-like objects’ incompletely filling 3-dimensional space. (The associated solvent fills the rest of the space.)

It was found that the SAXS intensity could be fitted well using the Debye model (Equation (1), [61]), which allowed *R_G_* to be evaluated. Results from individual specimens ranged from around 8 to 17 nm, consistent with the variation observed by Greving et al. [60] and appeared to remain constant between 25 and 55 °C. Since the variation of results from individual specimens were smaller (standard deviation, SD < 1.4 nm) than between specimens (SD = 2.7 nm), this may reflect differences between the original NSF samples [97], such as that caused by the naturally occurring Ca^2+^ and K^+^ contents [65], or other unidentified issues during the dilution process. Nevertheless, the value obtained from independent specimens was 12.5 ± 2.7 nm (average ± SD), in good agreement both with the results by Greving et al. [60] and expectations based on a protein of 5525 amino acids from the published sequences [63] in a random coil configuration under theta-conditions, with a monomer length of 0.4 nm [64], calculated using Equation (2) [62].

It should also be noted that at the concentration used, the values of *R_G_* obtained were consistent with the solution being ‘dilute’ (i.e., the protein coils were sufficiently separated that they did not overlap). As noted by Greving et al. [60] this criterion is essential in order to obtain the molecular dimensions from small-angle scattering measurements.

During heating, the scattering curves did not change significantly below 60 °C, suggesting that *R_G_* remained constant up to this temperature. Above this temperature, however, the intensity at low q increased, such that the plot (on double-logarithmic axes) became almost a straight line across the entire scattering range, as shown by the data at 65 °C in Figure 1a. While some curvature persisted, it was possible to fit the Debye model; the resulting values of *R_G_* are shown in Figure 1b. This rapidly ceased to be possible above 65 °C, however, which was consistent with aggregation of the protein chains in the diluted NSF.

It is interesting to note that these changes in the diluted NSF also coincided with thermally induced gelation of (undiluted) NSF at similar temperatures, as observed previously by rheology (above 60 °C) [9,12] and calorimetry (60–68 °C) [11,13,16]. This apparent insensitivity to concentration suggests that the configurational entropy of mixing, which constitutes a significant part of the Flory–Huggins model for polymer solubility [62,100], is not applicable to fibroin in NSF. Instead, it appears that solubility of fibroin in NSF is dominated by the strength of interaction between the protein and water (i.e., the interaction parameter term in the Flory–Huggins model).

### 2.2. Protein Coil Size by DLS

The size of protein chains in diluted NSF and their behaviour during heating were investigated by DLS. Although this method also uses scattering, DLS measures the autocorrelation of the scattered light over time at a fixed angle, from which the autocorrelation function and *R_H_* of the scatterers can be obtained [100,101,102,103]; thus, the results from DLS may be regarded as providing an independent check on the results from SAXS. It should be emphasised, however, that *R_H_* obtained by DLS depends on the diffusion rate of the molecules while *R_G_* obtained by SAXS or SANS represents the distribution of mass within an average molecular coil. Hence, for any given molecular geometry, the values of *R_G_* and *R_H_* should be related but may not be identical.

**Figure 2:**
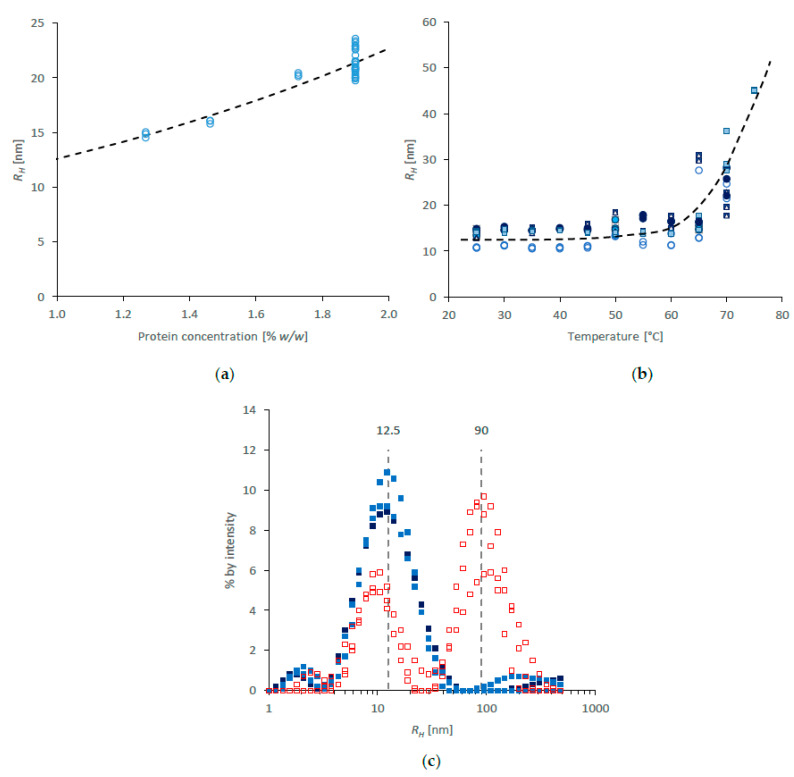
DLS data for diluted NSF solutions (**a**) Effect of concentration on (apparent) hydrodynamic radius, (**b**) change in *R_H_* with temperature, different colours and shapes of symbols represent different specimens; (**c**) size distributions extracted from data at 25 °C (in blue) and 70 °C (in red). The dashed lines in (**a**,**b**) serve only as guides for the eye.

DLS data for NSF diluted in water is shown in Figure 2. First, in order to check whether concentration affected the results, specimens were measured multiple times at the starting concentration (1.9% *w*/*w*, determined gravimetrically) and after further dilution with type 1 water. The results in Figure 2a suggested a small effect over the concentration range used, with further dilution producing smaller *R_H_* (Z-average values from around 21.4 ± 1.1 nm at 1.9% *w*/*w*, to around 15 nm at 1.3%). This result appears to fit with previous findings by Ochi, Hossain and coworkers [12,91,104], who assigned values between 12 and 20 nm to individual fibroin chains from different parts of the silk gland.

Some variation was seen between initial specimens at 25 °C (range: 10.8–14.9 nm; SD = 1.3 nm), similar to that observed using SAXS. During heating, the values of *R_H_* appeared to remain essentially constant below 60 °C, but increased rapidly above that temperature (Figure 2b)—also reminiscent of the SAXS data (Figure 1b). Thus, the thermal behaviour revealed by DLS corroborated that observed by SAXS. Using both techniques, the protein aggregation in dilute solution coincided with thermal gelation in NSF [9,11,12,13,16], thereby emphasising the apparent insensitivity of fibroin solubility to concentration.

Further examination of DLS data suggested that *R_H_* at room temperature was essentially distributed about a single mode of around 12.5 nm (as in Figure 2c), which may be ascribed to individual fibroin chains. Slight deviations from the baseline at lower and higher values of *R_H_* are not thought to be significant. In this respect, the DLS results presented here conflict with those presented earlier by Ochi, Hossain and coworkers [12,91,104], who reported significant populations with larger *R_H_* in freshly prepared fibroin solutions, which they ascribed to complexes containing multiple protein chains. Taken together with the rheology they reported [12], however, it appears that their preparation methods may have resulted in some premature fibroin gelation.

Heating above 60 °C caused the appearance of a second mode, centred around 90 nm (Figure 2c), consistent with aggregation of the fibroin. Interestingly, the residual population of individual chains appeared to show a slight reduction in *R_H_*, which may indicate a decrease in hydration as a precursor to aggregation.

### 2.3. Observing Silk Protein Hydration by Mid-IR Spectroscopy

**Figure 3:**
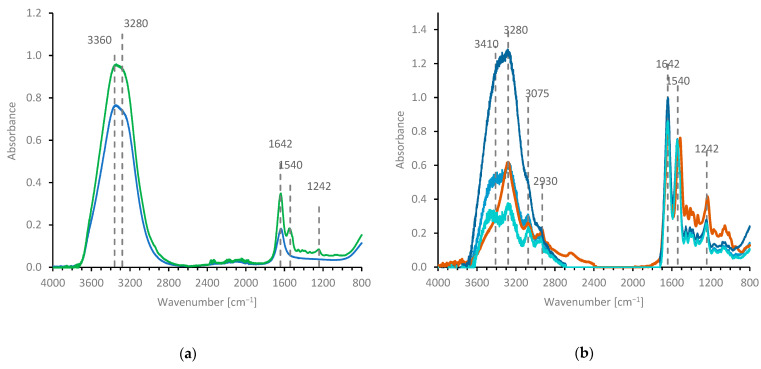
Mid-range IR data, collected using ATR: (**a**) NSF (green) and water (blue, scaled to match concentration in NSF); (**b**) dried fibroin film (brown) and difference spectra (NSF–water, various shades of turquoise) with the weighting factors for the water spectrum shown, then scaled to match the amide II peak intensity in the spectrum of the dry film.

Unsurprisingly, the IR spectra of NSF was dominated by water, as demonstrated in Figure 3. The molar ratio of water to peptide groups in NSF was around 14: 1 (for 23% *w*/*w* fibroin, using the average amino acid formula weight of 75.9 g mol^−^^1^, based on the published sequences [63]). Consequently, the strongest feature in the NSF spectrum (green curve) was the broad peak (between 2800 and 3800 cm^−^^1^) due to water (blue curve). Although this can be ascribed mainly to O-H stretching vibrations, the apparent simplicity of the water molecule belies the complexity of this absorbance band. The symmetric and asymmetric O-H stretching vibrations of water vapour occur at 3657 and 3756 cm^−^^1^, but these are shifted to lower frequency through strong H-bonding in the liquid state [105,106,107,108,109,110,111,112]. The subsequent overlap with the first overtone of the bending mode (fundamental around 1640 cm^−1^ in liquid water) then allows further contributions due to Fermi resonance [109,110,111,112]. Consequently, the assignment of individual bands in this region remains uncertain.

Only the strongest peptide bands (amide I, II and III, around 1642, 1540 and 1242 cm^−^^1^ [113,114,115]) were immediately obvious, although closer scrutiny revealed other small protein bands around 1000 to 1500 and 2800 to 3000 cm^−1^. While the problem of a weak spectrum being obscured by a stronger one could, in principle, be solved by acquiring high quality data and performing a careful subtraction, it was not possible to obtain a reliable spectrum for silk fibroin simply by subtracting a suitably weighted contribution due to pure water from the spectrum of NSF, as demonstrated in Figure 3b. Based on the expected composition of NSF (around 77% water), the difference spectrum (in turquoise) was weaker than the dry fibroin spectrum (in brown) below 1500 cm^−^^1^, but more intense above 3000 cm^−^^1^. Consequently, the amide A and B (around 3280 and 3075 cm^−^^1^, due to Fermi resonance of N-H stretching and the first overtone of the amide II bending mode) and C-H stretching bands (around 2980 cm^−^^1^) of the fibroin remained largely hidden within the broad absorbance envelope of water. A somewhat better match was obtained after subtracting a larger water contribution (0.88). In this case, the amide A and B and the C-H stretching peaks were clearer and more closely matched the peak heights in the dry fibroin spectrum, although residual intensity around 3410 cm^−^^1^ remained in this spectrum. Moreover, subtracting an even larger water contribution (0.92) reduced the height of the amide A peak unreasonably (relative to the C-H stretching peaks), while excess absorbance still persisted above 3410 cm^−^^1^.

The problems associated with subtracting a water spectrum were observable more clearly using NMAc, a secondary amide commonly used as a model for the peptide group. Since NMAc is miscible with water in all proportions, it was quite simple to explore amide concentrations much higher than in NSF. Conveniently, the formula weight of NMAc (73.1 g mol^−^^1^) is also close to the average for an amino acid repeat unit in fibroin (75.9 g mol^−^^1^), so direct comparisons of concentrations may be made.

**Figure 4:**
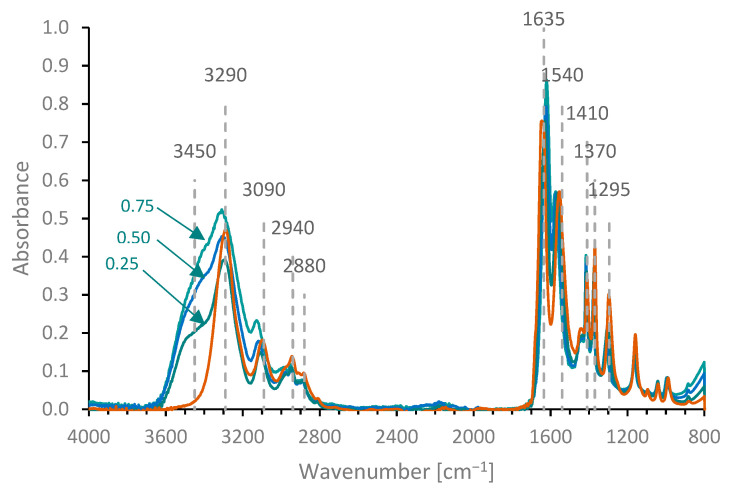
ATR-IR spectra of pure (liquid)NMAc (brown) and aqueous (25, 50 and 75%) NMAc solutions (turquoise), after subtraction of a pure water spectrum, using the weightings expected (as shown) and scaled to match the amide II peak intensity in the dry NMAc spectrum.

For each of the compositions investigated (25, 50 or 75% *w*/*w* NMAc), subtracting a water spectrum based on the solution concentration resulted in reasonable, though not perfect, matches to the pure NMAc spectrum below 3290 cm^−^^1^, but left a clear excess absorbance around 3450 cm^−^^1^ (Figure 4). This improved for more concentrated NMAc solutions as the spectral contributions due to water decreased; nevertheless, considerable excess absorbance remained even for a 75% NMAc solution (corresponding to a weighting of 0.25 for the subtraction of the water spectrum).

Various ‘practical’ issues could affect the spectra acquired by ATR [114,115,116,117,118,119,120]; for example, band shapes and positions can be distorted due to insufficient differences in refractive index between the specimen and ATR element. Differences in refractive index between specimens would affect the ‘sampling depths’ (i.e., penetration of the evanescent electric field of the IR photons). Furthermore, phase separation in the NSF specimen could allow composition changes (e.g., water enrichment) near the ATR element—although this can be ruled out for the NMAc solutions. Nevertheless, these effects do not appear to provide a convincing explanation for the difficulties in obtaining a difference spectrum matching that of dry fibroin or NMAc (in particular, the persistence of excess aborbance above 3410 cm^−^^1^).

A more likely explanation involves interactions between the solute (NMAc or fibroin) and water. A considerable body of work [113,114,115,121,122,123,124,125,126,127,128,129,130,131,132,133,134,135,136,137,138,139,140,141,142,143] has explored the hydration of amides; much of this has been directed towards understanding how it affects the vibrational spectrum of the amide, but a corresponding effect of the amide on the bonding and, consequently, the spectrum of water can be expected [123,124,125,126,127,128,133]. In particular, it appears that these interactions increased the absorbance due to water in the region of 3410 to 3450 cm^−^^1^, since a simple shift in peak position on its own would result in a corresponding negative band after subtracting the pure water spectrum. That is, O-H stretching bands of water interacting with amides are more intense and at higher wavenumber, compared with pure water. This concurs with recent observations of stretching bands between 3468 and 3536 cm^−^^1^, for water H-bonded to amides, isolated in inert media [126]. It is also consistent with the suggestion of stronger H-bonding between peptide groups and water, compared with pure water [123,124,125,129]. It is not clear, however, whether this increased intensity can be attributed to a specific vibration, or whether it originated from more complex interactions within the amide-water system. The amide group can form two H-bonds (as proton acceptor) via the amide oxygen, and another (as proton donor) via N-H [123,127,129,131]. Hence, around 21% (3 out of 14) of the water molecules in NSF may be directly H-bonded to the peptide. Due to the extensive H-bonding in liquid water [105,106,107,108,109,110,111,112], however, the majority of the other water molecules in NSF may also be affected by the peptide groups.

The absorption spectrum is likely to be further complicated, however, through extensive coupling and resonance in the water-amide system:

- Fermi resonance between O-H stretching and the first overtone of the water bending mode [105,106,107,108,109];
- Fermi resonance between N-H stretching and the first overtone of the amide I band (giving rise to the amide A and B bands) [113,114,115];
- sufficient strength of H-bonding between water and amide groups to allow resonance between the amide I (mainly C=O stretch) and the water bending mode, facilitating rapid transfer of vibrational energy between the protein and its hydration shell [121,122,128];
- possibly also resonance between O-H stretching bands of water and the amide A and B bands.

Thus, the amide-water system appears to be exquisitely set up to allow the rapid transfer of vibrational energy.

The increased absorbance above 3400 cm^−^^1^ may be regarded as complementary to the changes observed in the spectra of the solutes: the amide II and III bands of fibroin (Figure 3b) and NMAc (Figure 4) were at higher wavenumber in the solutions, while the amide I band of NMAc in solution was at lower wavenumber compared with the dry state. Conversely, spectral bands associated with other groups in protein or NMAc (e.g., C-H stretching or bending) did not change significantly between solution and dry states, which emphasises the role of H-bonding in these systems.

### 2.4. Changes in Hydration during Heating

The amide II and III bands (around 1540 and 1242 cm^−^^1^, asymmetric and symmetric resonance combinations of >N-H bending and C-N stretching [113,114,115]) are well separated from the water bending band, hence, these bands should be free from resonance. They are still affected by coupling to the hydration shell, however, and may be used to investigate changes in solvation with temperature.

**Figure 5:**
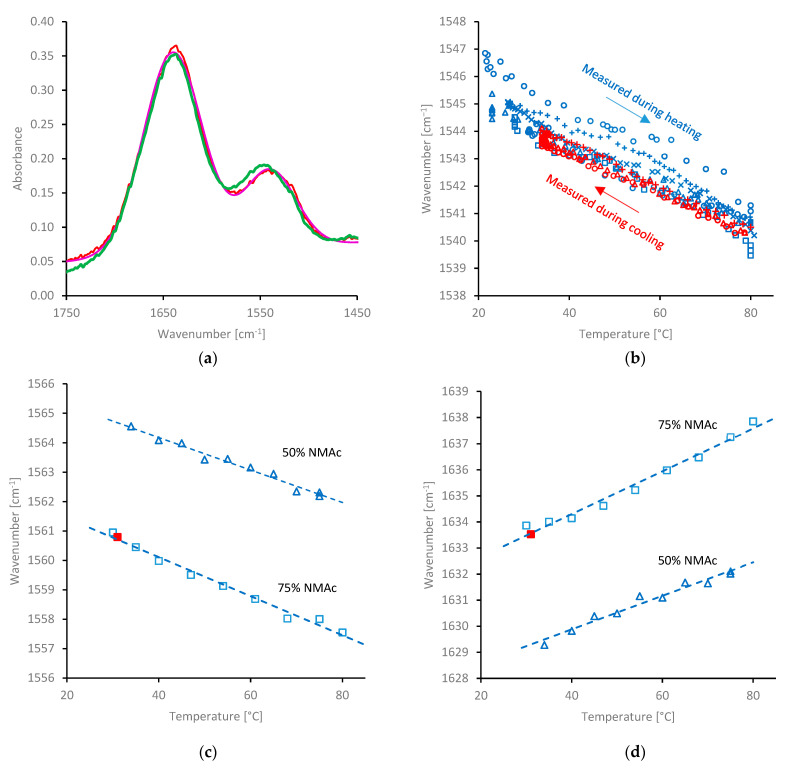
Effect of temperature on the IR spectra of NSF and NMAc: (**a**) amide I and II region for NSF measured at 27 (green) and 81 °C (red), with a Gaussian model fitted to the 81 °C data (magenta); (**b**) plot of amide II peak position vs. temperature for NSF; the different symbols represent different NSF specimens, blue points were measured during heating from room temperature, red points were measured during cooling from 81 °C; (**c**): amide II and (**d**) amide I peak positions for 50 and 75% *w*/*w* aqueous NMAc solution; blue open points were measured during heating, the red filled points were measured after subsequent cooling of the 75% NMAc solution.

The IR spectra between 1450 and 1750 cm^−^^1^ for NSF, measured at 27 (green) and 81 °C (red) are shown in Figure 5a. Little change was observable for the band around 1640 cm^−^^1^, which included absorbance due to both the amide I of the protein and the water bending mode. Consistent with previous observations [105,106], however, the water bending band in pure water changed little over this temperature range, which may have obscured any potential change in the amide I position. Consequently, no further attempt was made to analyse movement in the amide I peak for NSF specimens.

By contrast, a clear shift of the amide II was evident, from around 1545 cm^−^^1^ at 27 °C, to around 1540 cm^−^^1^ at 81 °C. The change in the amide II position was examined more closely by fitting a Gaussian model to the IR absorbance data:

(3) $$S\left(\nu \right)=\;\left[a+m\nu \right]+\underset{i}{\sum }A_{i}.\exp\left(-{\left(\frac{\upsilon -\nu_{i}}{2\Delta_{i}}\right)}^{2}\right)$$

where *A_i_*, *ν_i_* and Δ*_i_* represent the intensity, centre position and breadth of the *i*-th component, *ν* is the wavenumber and the first term in square brackets (with *a* and *m* constant) is a linear baseline approximation under this part of the spectrum. Although both peaks may contain several contributions, due to overlapping vibrations and different conformations or H-bonding environments, it was found that using only two Gaussian components (i.e., one centred around 1640 cm^−^^1^ and the other for the amide II) generally produced the most reliable results. Thus, the changes in the amide II peak position were evaluated (Figure 5b).

The peak position at room temperature appeared to vary slightly (from around 1544 to 1547 cm^−^^1^) between NSF specimens. This variation was considerably larger than could be ascribed to uncertainty in the peak fitting procedure; repeated attempts on the same data generally converged to within 0.1 cm^−^^1^. Close examination of the example in Figure 5a also shows that the model curve (in magenta) closely matched the experimental data (exemplified using the data at 81 °C, in red). Thus, the variations in peak positions appear to reflect real differences between specimens, although it is uncertain whether they were due to the conditions of the silkworms used or as a result of how the specimens were extracted. (The reader may note that unexplained variations between specimens were also observed by SAXS and DLS).

In all cases, the amide II peak moved to lower wavenumber as the temperature was increased. Moreover, starting at higher temperatures, the peak moved back towards a higher wavenumber as the specimens were cooled (shown in red). This demonstrates that the effect was not merely due to the time since the specimens had been dissected from the silkworms and placed on the ATR device. It may also be noted that the locus of the data during cooling fell along the lower limit of the data during heating, which may reflect that these specimens had undergone changes associated with gelation during the initial heating.

Further evidence of the effect of temperature on the amide band positions was obtained by examining the spectra of other secondary amides. Movement of the amide II peak to lower wavenumber at higher temperatures and returning to the original position on cooling was observed for 50 and 75% *w/w* NMAc solution (Figure 5c); similar changes were also found with 25% *w/w* NMAc solutions and 33% *w/w* pNiPAm solutions (data not shown). Movement of the amide III band (also involving C-N stretching [113,114,115]) to lower wavenumber at higher temperature was also observed, consistent with the changes in amide II position. This was not analysed in more detail in the present work, however, due to the relatively small intensity of this band and its proximity to other bands in the fibroin or NMAc spectra. Movement of the amide I peak was also observable in the more concentrated (50 or 75% *w/w*) NMAc solutions. Curve fitting indicated movement to higher wavenumber during heating, returning to the original position on cooling, i.e., opposite to the behaviour of the amide II and III bands (Figure 5d).

**Figure 6:**
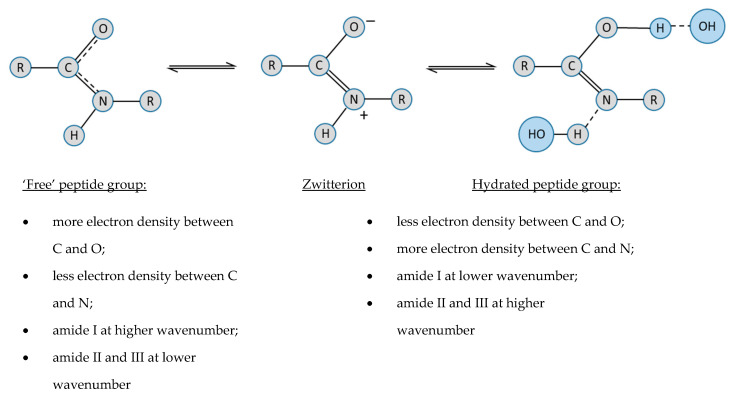
Valence band formalism to describe the changes in electron density around the peptide group in the zwitterionic form (centre) or due to hydration (right): R indicates a methyl group in NMAc or the rest of the chain in a protein, solid lines indicate complete bonds, dashed lines indicate partial and H-bonds.

The differences in the amide (I, II and III) peak positions in the presence of water or during heating can be explained by considering the effects of H-bonding on the electron density distribution across the peptide group (Figure 6) [103,104,105,126,129,130,131,132]. Using valence bond formalism, there is significant overlap between the electron lone pair on the N atom and the C=O π-bond. This delocalisation gives a significant double-bond character across the entire amide group, which explains its planar geometry and the significant energy barrier (around 80 kJ mol^-1^) that impedes rotation about the C-N bond [132,133]. Hydration changes the electron density within the amide group, corresponding to an increase in the double-bond character between C and N, but a decrease between C and O. Amunson and Kubelka [134] suggested a different hypothesis in which a solvent of higher dielectric strength favours the zwitterionic form of the peptide group. In either case, the amide I band (mainly due to the carbonyl stretching vibration) moves to lower wavenumber and the amide II and III (both combination bands involving the C-N stretching vibration) move to a higher wavenumber.

Consequently, the changes observed in amide I, II and III band positions with temperature provide strong indication that interactions between the amides and water become weaker as the temperature is increased. Moreover, it should be emphasised that this occurred in the absence of a phase change with NMAc solutions, or before any phase change in NSF (i.e., while still below 60 °C). Hence, the decrease in hydration at higher temperatures appears to be a precursor to, rather than a consequence of gelation.

### 2.5. Observing Changes in Amide and Peptide Hydration by Near-IR Spectroscopy

Due to the intensities of bands in the mid-range IR (400–4000 cm^−^^1^), spectroscopy of aqueous materials is constrained to very thin specimens (<20 µm) or reflectance methods. By contrast, the combination and overtone bands in the NIR range (>4000 cm^−^^1^) tend to be weaker, such that transmission measurements on thicker specimens (up to millimetres) are possible [144,145,146,147,148,149,150,151,152,153,154,155,156]. Thus, NIR can be very useful for studying aqueous solutions of proteins, peptides or models.

**Figure 7:**
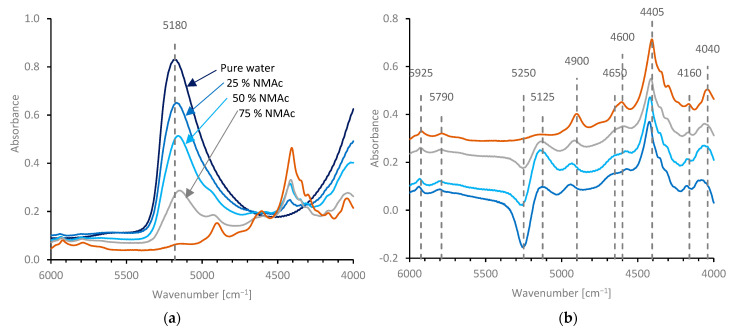
(**a**) NIR spectra for dry NMAc (brown) and aqueous NMAc solutions of the compositions indicated, collected in transmission geometry; (**b**) difference spectra after subtracting the expected water contributions and scaling to match the intensity of the peak around 4405 cm^−1^. The same colours are used for the specimens in both graphs and the plots in (**b**) have been off-set vertically for ease of viewing.

NIR spectra (from 4000 to 6000 cm^−1^) for water, NMAc and selected aqueous solutions are shown in Figure 7a. These spectra were dominated by a (relatively) strong band (at 5180 cm^−1^ in pure water), which can be ascribed to a combination of O-H stretching and bending [152,153,154]. (It should be noted that there was a molar excess of water in each of these solutions; a 25% *w*/*w* NMAc solution corresponds to a molar ratio of 12:1, while 75% *w/w* NMAc corresponds to a molar ratio of 1.35:1 water to amide). It may also be noted, however, that this peak did not coincide with the sum of the stretching and bending peak positions of pure water (3360 + 1642 = 5002 cm^−1^), possibly because the former contains several components [105,106,107,108,109,110,111,112], of which the highest frequency vibration (around 3580 cm^−^^1^, by peak fitting) dominates the combination.

As this region is essentially free from NMAc bands, movement of the water band to lower wavenumber with increasing NMAc concentrations (to 5140 cm^−^^1^, in 75% *w/w* NMAc) could be clearly observed. This may be ascribed to progressively stronger or more extensive H-bonding with the amide, causing a bathochromic shift of the O-H stretching fundamental or through the suppression of the highest frequency modes.

Difference spectra (NMAc solutions minus water) are shown in Figure 7b. The changes in the position of the water band with NMAc concentration caused a mismatch in the subtraction, which produced a negative peak around 5250 cm^−^^1^ and a positive peak around 5125 cm^−^^1^ in the difference spectra. The NIR spectrum of NMAc (dry or in solution, after subtracting the water spectrum) was dominated by a peak around 4405 cm^−^^1^, which appears to be composed of several absorbance bands. Other smaller peaks were observed between 4000 and 6000 cm^−^^1^, as indicated in Figure 7b. By comparison with the fundamental bands shown in Figure 4, the majority of these appear to be combinations involving vibrations of the amide group, although the two bands at 5790 and 5925 cm^−^^1^ may be overtones of C-H stretching modes. Consistent with this, the majority of the bands (i.e. excepting those at 5790 and 5925 cm^−^^1^) appeared to change position quite considerably (by up to 40 cm^−^^1^, for the band around 4900 cm^−^^1^) between dry NMAc and aqueous solution, as shown in Figure 7b. In most cases, the peaks moved to a higher frequency in aqueous solution, although the peak at 4600 cm^−^^1^ appeared to move to a lower frequency.

**Figure 8:**
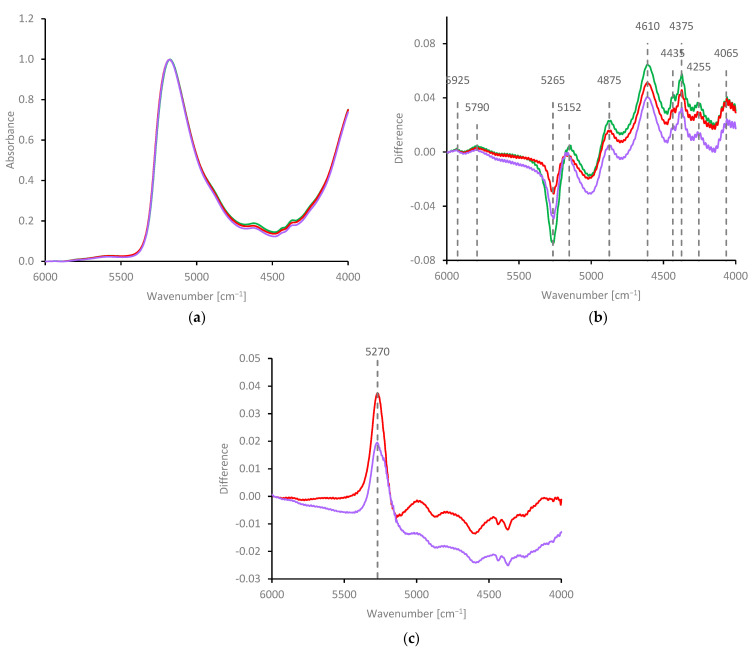
NIR spectra for silk fibroin: (**a**) NSF collected fresh from a silk gland and after gelation by freezing or heating; (**b**) difference spectra, from the data in (**a**) after subtracting a water spectrum; (**c**) difference spectra for, gelled specimens after subtracting the fresh NSF spectrum. All spectra were recorded at room temperature (22 °C) and the same colours are used throughout for specimens of fresh NSF (green) and gelled specimens produced by freezing (mauve, −28 °C for 30 min) or heating (red, 82 °C for 5 min).

The water band also dominated the NIR spectrum of NSF (Figure 8a); however, subtraction of a water spectrum produced a reasonably good spectrum for native fibroin, as shown in Figure 8b. This spectrum appeared essentially identical to that for redissolved fibroin, as reported previously by Mo et al. [155,156]. Rough similarities could also be seen between the spectra for fibroin and NMAc (shown in Figure 7b), although the peaks were in slightly different positions. The most significant difference was that the small peaks at 4600 and 4650 cm^−^^1^ in the NMAc spectra were replaced or obscured by a (relatively) large peak at 4610 cm^−^^1^ in the fibroin spectrum, which may originate from combinations of C-H stretching and alkyl bending modes of the various amino acid side groups [155].

Gelation by freezing (30 min at −28 °C, mauve) or heating (5 min at 82 °C, red) produced some small changes in the water band, between 5000 and 5500 cm^−^^1^. Specifically, the peak around 5152 cm^−^^1^ appeared to move to higher wavenumber, while the negative peak around 5265 cm^−^^1^ increased for the gelled specimens. These features can both be ascribed to the mismatch in water subtraction from the NSF spectra; hence, they appear to indicate changes in hydration associated with gelation.

These changes are revealed more clearly by examining the difference spectra (gelled minus fresh NSF, Figure 8c). In both cases, a single (positive) peak around 5270 cm^−^^1^ was produced, which suggests that the absorbance due to water was slightly stronger and at higher wavenumber in the gelled specimens, compared with fresh NSF. To the best of our knowledge, this is the first time changes in hydration have been revealed by comparing NIR difference spectra of closed systems at the same temperature, before and after gelation. Nevertheless, the inferences are similar to those made by many other workers. For example, displacement of the hydration shell during gelation has also been suggested by Mo et al. [156] based on NIR observations of water loss during heating.

Supporting evidence for the displacement of the hydration shell can also be found in a previous publication by Mapelli et al. [157]. Using magnetic resonance imaging (NMR microscopy), it was found that as NSF in an excised silk gland gradually gelled at room temperature, the water signal became more intense. Although those authors did not explore the phenomenon in more detail, it is consistent with an increase in water mobility, corresponding to slower (transverse, *T*_2_) nuclear relaxation, such that a stronger water signal persisted in their imaging method.

### 2.6. Cloud-Point and Aggregation Measurements in Salt Solutions

Thus far, we have demonstrated that the solubility of fibroin (in NSF or diluted solutions) appears unaffected by concentration, but depends on the strength of interaction between water and the peptide groups. Furthermore, a decrease in the interactions between water and amide groups during heating precedes NSF gelation. In general terms, this suggests that hydration of the fibroin is enthalpically favoured around room temperature, but is entropically disfavoured, such that it becomes thermodynamically unstable above a threshold temperature (around 65 °C, based on observations presented here and previously [9,16]). Moreover, if NSF is frozen, ice forms a lower energy phase, which draws water out of the fibroin hydration shell. In either case, loss of the hydration shell from the fibroin allows it to seek new intermolecular interactions, leading to protein aggregation and gelation. An embryonic version of this conjecture was presented previously [9]; in order to test it more thoroughly, however, the thermodynamics controlling hydration and solubility are explored in this section.

**Figure 9:**
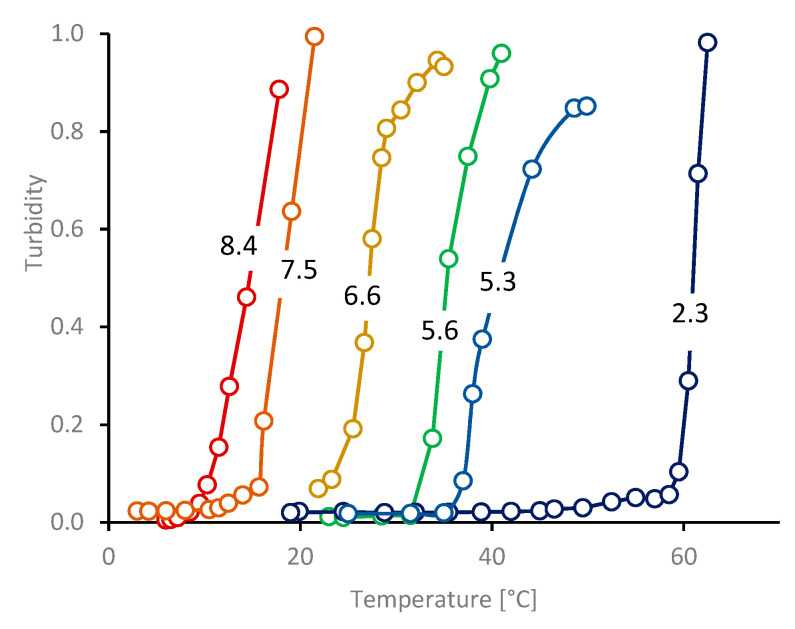
Representative examples of turbidity data vs. temperature, based on diluted NSF in NaCl solutions of the salt concentrations (% *w*/*w*) shown on the graphs.

Representative turbidity data for diluted NSF (ca. 0.1% *w*/*w* protein) in aqueous NaCl solutions are shown in Figure 9. Similar results were also obtained with KCl solutions (not shown). These salts were selected because they lie towards the centre of the Hofmeister series [158,159,160,161,162] and are not expected to show any chemical affinity for the protein. In particular, monovalent cations avoid any possibility of bridging between carboxylate groups [65,66,67], which could affect aggregation and turbidity measurements.

The turbidity is related to decreases in transmitted light, due to scattering from inhomogeneities in the dilute solution [98,100,163,164,165,166,167].

(4) $$\tau \left(\lambda \right)=Q\left(\lambda \right)\cdot \frac{32\pi^{3}{\left\{n_{0}\left(\lambda \right)\right\}}^{2}}{3N_{A\;}\lambda^{4}}\cdot cM{\left(\frac{dn\left(\lambda \right)}{dc}\right)}^{2}$$

where *N_A_* is Avogadro’s constant and *n_0_* is the refractive index of the solvent. The ‘transmittance dissipation factor’ *Q(λ)* is a dimensionless number that depends on the size of the scattering particles relative to the wavelength, and is between 0.99 and 1 if the scatterers are small relative to the wavelength of light, which appears to be valid for diluted fibroin (*R_G_* ≃ 12 nm, *R_H_* ≃ 12.5 nm, based on results from this work) observed using visible light (λ = 500 to 700 nm). The protein concentration (*c*, by weight) remained constant during each experiment. Furthermore, significant changes in *dn/dc* (implying changes in chemical composition) or *Q(λ)* (only expected after increased particle size towards λ) are not expected to affect the initial onset of turbidity, although they cannot be excluded following more extensive aggregation. Hence, the initial increases in turbidity at the cloud points can be ascribed to increases in the masses (*M*) of the scattering particles, consistent with protein aggregation revealed by SAXS and DLS. These data showed a clear ‘salting out’ effect, with more concentrated salt solutions producing turbidity at lower temperatures.

According to our hypothesis, the driving force behind the stability of the hydration shell can be considered in terms of the chemical potential of water:

(5) $$\mu_{w}=\;{\left(\frac{dG}{dn_{w}}\right)}_{T,P,x}$$

that is, the change in free energy per mol of water, keeping temperature, pressure and composition constant. Thus, the hydration shell remains stable while the chemical potential of the associated water is lower than that of the bulk aqueous phase, but becomes unstable if its chemical potential is higher. The addition of salts (or other solutes) reduces the chemical potential of water, which can be evaluated through the reduction in its vapour pressure (*p*):

(6) μw= μw#+RT·ln(pp#)

where *R* is the gas constant and the # superscript indicates the value for pure water. This calculation becomes particularly simple if non-volatile solutes are used, as only the water contributes to the vapour pressure (otherwise, the partial pressures of water and the solute should be evaluated). Moreover, several studies reporting the vapor pressure over salt solutions are available [168,169,170,171,172]; consequently, it is a relatively simple matter to obtain aqueous solutions of diluted NSF in which the chemical potential of the bulk water phase can be calculated.

**Figure 10:**
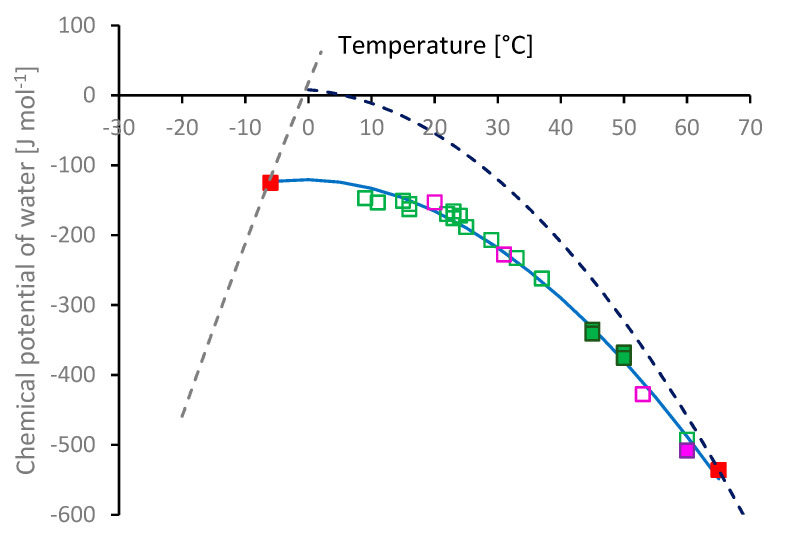
Chemical potential vs. temperature for ice (grey dashed, Equation (7a)), liquid water (blue dashed, Equation (7b)), cloud points in salt solutions (open squares) and the aggregation onset points observed by DLS or SAXS (filled squares). Green points were obtained for NaCl solutions; magenta indicates KCl solutions. The gelation points at high and low temperature, observed by rheology (red), are assumed to indicate where the chemical potential of the hydration shell is equal to that of water or ice. The uncertainty in the experimental data is expected to be similar to the sizes of the symbols. Subsequently, an estimate for the chemical potential of the hydration shell (blue line) was obtained by fitting Equation (8) to the turbidity and aggregation point data.

The chemical potential of water in each salt solution was calculated using Equation (6), based on published vapour pressure data. Graphs of chemical potential against cloud-point temperature are shown in Figure 10. The curves for ice (grey dashed line) and water (blue dashed line) were obtained using published data [173,174], relative to the values at the triple point of water (273.16 K at 611.657 Pa). For the present purposes, it was found that these properties could be fitted adequately using quadratic expressions:

(7a) $$\mu_{ice}\left(T\right)=10.286+22.186T-0.0717T^{2}$$

(7b) $$\mu_{water}\left(T\right)=8.038-0.7842T-0.1167T^{2}$$

for the chemical potential in J mol^−^^1^ and temperature in °C.

The temperatures where NSF was found to gel through freezing (at −6 °C) or heating (65 °C) are marked (in red) on the ice or water curves. Close agreement was found between gelation at elevated temperature observed by rheology [9] and the denaturation endotherm shown by differential scanning calorimetry (DSC) [16]. In both cases, the processes began around 60 °C and achieved their maximal rates around 65 °C. Less good agreement was found for the low temperature gelation; however, rheology indicated freezing and gelation around −6 °C, while ice melting was only observed by DSC after the specimen had been cooled below −12 °C. In this case, the value indicated by rheology (i.e., by an increase in dynamic moduli or a decrease in the phase angle below 45°) is thought to be more relevant, as it can be ascribed to a direct observation of the onset of ice formation and protein aggregation. Thus, the gelation temperature for NSF by freezing is taken from observations reported previously [9], which we have subsequently corroborated (data not shown). The lower value indicated by DSC corresponds to the formation of sufficient ice in a form that gave a discernible melting endotherm. Close examination of the data presented by Holland et al. [16] showed considerable depression of the melting point, with the endotherm (Figure 4b in Holland et al. [16]) starting well below 0 °C (around −10 °C). That may have been due to the Gibbs–Thompson effect [175,176,177], with ice crystals constrained to nanoscopic size by the porosity of the fibroin gel. Thus, the ice formed during the earliest stage of freezing may have remained undetected by DSC, due to its limited amount and broadened melting endotherm.

Cloud and aggregation points for diluted fibroin in salt solutions are also marked (Figure 10, green for NaCl, magenta for KCl solutions). Consistent with expectations based on the hypothesis described above, it was found that all these various points (for NSF and diluted fibroin) lay along a single curve.

A thermodynamic model for the chemical potential of the hydration shell was developed, containing only three adjustable parameters:

(8a) $$\mu_{hyd}\left(T\right)=\;\Delta H_{hyd}\left(T\right)-T\cdot \Delta S_{hyd}\left(T\right)$$

where:

(8b) $$\Delta H_{hyd}\left(T\right)=\;\Delta H_{hyd}^{0}+C_{P}\cdot \left(T-T_{0}\right)$$

(8c) $$\Delta S_{hyd}\left(T\right)=\Delta S_{hyd}^{0}+C_{P}\cdot \ln\left(\frac{T}{T_{0}}\right)$$

The constants $\Delta H_{hyd}^{0}$ and $\Delta S_{hyd}^{0}$ represent the changes in enthalpy and entropy from pure water to the fibroin hydration shell at an arbitrary reference temperature (*T_0_*, chosen as 0 °C). Further changes in enthalpy and entropy of water in the hydration shell, away from *T_0_*, are described by the terms involving the heat capacity at constant pressure (*C_p_*) in Equations (8b) and (8c). For simplicity, *C_p_* of water in the hydration shell was assumed to be a constant in the present model, although this may not be strictly true as the values for liquid water are known to change slightly across the relevant temperature range (76.01 J mol^−^^1^ K^−^^1^ at 0°C, to a local minimum 75.29 J mol^−^^1^ K^−^^1^, around 37 °C, and 75.41 J mol^−^^1^ K^−^^1^ at 62°C [173]).

The constants were evaluated by fitting the model to the data (minimising deviations, using ‘Solver’ in Microsoft Excel), giving $\Delta H_{hyd}^{0}$ = −69.7 J mol^−^^1^, $\Delta S_{hyd}^{0}$ = 0.2 J mol^−^^1^ K^−^^1^ and C_p_ = 57.9 J mol^−^^1^ K^−^^1^. Remarkably, in spite of the evident simplicity of this model, it was found that the corresponding plot of the chemical potential for the hydration shell fitted the data closely, as shown by the blue solid curve in Figure 10.

## 3. Discussion

From a wide-ranging set of experiments, we have demonstrated several important features regarding the solubility of B. mori silk fibroin and its gelation.

(i) In dilute solution, the protein coil exhibits a three-dimensional Gaussian random walk configuration, typical of a polymer in solution under theta conditions. This implies an approximate equivalence between the strengths of monomer-monomer and monomer–solvent interactions. Moreover, while it was not possible to observe the coil geometry by SAXS (or SANS [60]) at higher concentration (the interpretation of scattering data required non-overlapping coils), the configuration determined at low concentration was consistent with previous NMR results [46,47,48,49] and rheology [9,99,178], which demonstrated that the protein in NSF behaved as a typical polymer in solution, albeit slightly modified by its natural propensity to form transient ‘sticky’ ionic interactions [66,67].
(ii) Crucially, the combination of random coil geometry and uniform dynamics (from NMR studies [46,47,48,49]) implies similar levels of hydration across the entire fibroin coil, which precludes any explanation of gelation through interactions between hydrophobic regions.
(iii) The temperature at which the protein came out of solution (causing gelation of NSF or aggregation and turbidity in diluted solutions) appears to be independent of concentration. In terms of the Flory–Huggins theory [62,100], this implies that the configurational entropy of mixing is negligible, with solubility being dominated by the strength of monomer–solvent interactions (i.e., the interaction parameter, χ). It should be noted that, contrary to earlier descriptions, it is known that χ is not a constant describing purely enthalpic interactions, but varies with temperature and includes both entropic and enthalpic contributions [179,180,181,182].
(iv) Coupling and resonance between vibrations of water and peptide groups affect much of the mid-range IR spectrum. Nevertheless, changes in amide band positions of fibroin in NSF and other amide models demonstrated that the strength of interaction between water and peptide groups decreases with increasing temperature. Moreover, this occurred as a precursor to—rather than a consequence of phase separation.
(v) Subtle changes in hydration associated with gelation of NSF (by heating or freezing) were also demonstrated by NIR.
(vi) Based on our hydration shell hypothesis, a thermodynamic model was developed that closely fitted the various data from gelation (of NSF) and aggregation or turbidity measurements (of diluted fibroin).

**Figure 11:**
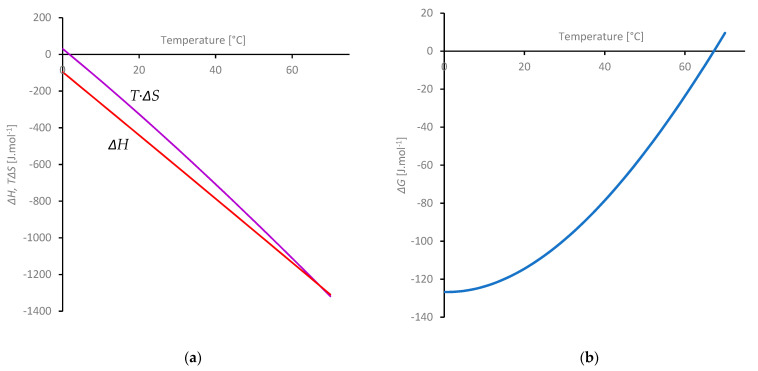
Thermodynamic values for the fibroin hydration shell, relative to free water, derived from the thermodynamic model (Equations (8a)–(8c)) fitted in Figure 10. (**a**) Enthalpic (Δ*H*, red) and entropic (*T*·Δ*S*, purple) components; (**b**) Gibbs free energy change is the difference (ΔG = ΔH−T·ΔS) between the plots in (**a**).

The thermodynamic factors driving desolvation of the fibroin (from 0 to 70 °C) were obtained from the differences between the values given for the hydration shell (by Equation (8)) and the corresponding values for pure (liquid) water; the results are shown in Figure 11. The values of Δ*H* and *T*·Δ*S* (Figure 11a) are both negative across (most of) this temperature range, consistent with the hydration shell being enthalpically favoured, but entropically penalised relative to free water. Both terms increase in magnitude with temperature. Moreover, the plots are almost parallel, producing a relatively small Gibbs free energy change as the difference between them (ΔG= ΔH−T·ΔS). This approximate balance between Δ*H* and *T·*Δ*S* is commonly known as ‘enthalpy-entropy compensation’. Similar phenomena have been observed in a wide range of systems [183,184,185,186,187,188], although a certain amount of controversy remains about whether it results from a deeper underlying mechanism. Particular insight has been provided by Dunitz [189] who suggested that for interactions between molecules (i.e., typical of solvation), stronger attraction (i.e., more negative Δ*H*) correspond to deeper, narrower potential wells with fewer accessible vibrational energy levels (causing more negative Δ*S*).

As a consequence, the resulting Δ*G* (Figure 11b) is negative across most of this range, consistent with a thermodynamically stable hydration shell (by up to around 128 J mol^−^^1^ at 0 °C). The stability decreases as the temperature is raised, however, and Δ*G* becomes positive above about 68 °C, corresponding to the hydration shell becoming unstable and being replaced by H-bonds between peptide groups, leading to gelation at elevated temperature.

Our model predicts an enthalpy penalty of around 1.27 kJ mol^−^^1^ of water at the gelation temperature. Even though free energy renders the hydration shell unstable, enthalpy is still required to release the water from the fibroin. We suggest this is the origin of the denaturation endotherms observed by DSC [13,16].

Previous DSC studies of B. mori NSF suggested a value around 1.8 J g^−^^1^ (of protein) for the denaturing endotherm [16], while slightly higher values (2.1 to 3.3 J g^−^^1^) were reported for fibroin from several wild silkworm types [13]. In view of the protein compositions, these correspond to relatively small values around 140 to 250 J mol^−^^1^ of amino acids. It seems likely, however, that the endothermic peaks observed by DSC were partially obscured by several simultaneous exothermic events (e.g., aggregation and crystallisation); consequently, they may not provide a useful estimate of the hydration enthalpy. Indeed, Hu et al. [190] observed exothermic crystallisation peaks during thermal analyses of silk protein films. Instead, we suggest that various DSC studies of bovine or human serum albumin [191,192] or hen’s egg ovalbumin [193] may provide more reasonable indications, giving enthalpy values equivalent to 1.76 to 2.08 kJ mol^−^^1^ of amino acids. Thus, the Δ*H* estimated by our model is consistent with gelation being initiated by the displacement of around 1.5 molecules of water per amino acid residue. In view of the assumptions and approximations used, this is encouragingly close to the expectation that two molecules of water should be displaced from each peptide to allow β-sheet formation (with the third water and amino acid side groups being accommodated between the sheets [194,195,196]).

Further insight can be gained by comparing the enthalpy released (Δ*H_solution_*) when NMAc is dissolved in water. This involves replacing the interactions between NMAc molecules with H-bonds to water, which represents the converse of fibroin gelation. Values of Δ*H_solution_* around −3.84 kJ mol^−^^1^ for crystalline [137,197,198], or −13.2 kJ mol^−^^1^ for liquid NMAc [199,200] have been reported at 25 or 30 °C, the difference (9.36–10.11 kJ mol^−^^1^) being the heat of fusion, Δ*H_f_* [201,202]. Moreover, extrapolating from the data published by Kreis and Wood [201] suggests Δ*H*_solution_ = −9.45 kJ mol^−^^1^ for NMAc at 68 °C.

It is generally accepted that 2° amides (i.e., NMAc and peptide groups) can form 3 H-bonds: two involving the oxygen as proton acceptor and a third involving >N-H as proton donor [123,127,129,131]. Modelling [129,130] suggests that the former are (60 to 90%) stronger than the latter. As β-sheet formation only involves two H-bonds between peptide groups, fibroin gelation may only require displacement of the water bonded to >N-H and one of the water molecules on the oxygen. Using these assumptions gives a rough estimate of Δ*H* around 5.8 kJ mol^−^^1^ per peptide, equivalent to 2.8 kJ mol^−^^1^ of water. Again, in spite of the obvious limitations of this estimate, including possible stereochemical restrictions on the hydration of a peptide compared with NMAc, the proximity to the value of Δ*H* suggested by our model (1.27 kJ mol^−^^1^) is encouraging.

Our model shows how Δ*S* and Δ*H* for the hydration shell change with temperature. A more fundamental picture emerges, however, by considering the difference in heat capacity between bulk water and the hydration shell. The specific heat capacity of water (4.19 kJ kg^−^^1^ K^−^^1^) is considerably higher than many other common liquids [203,204]. On a molar basis, this is equivalent to around 75.7 J mol^−^^1^ K^−^^1^ [173], which is considerably higher than the value estimated by our model for the hydration shell (57.9 J mol^−^^1^ K^−^^1^).

By definition, heat capacity measures the change in energy with temperature. At constant pressure:

(9) $$C_{p}\left(T\right)=\;\frac{dH\left(T\right)}{dT}$$

On a molecular level, the heat capacity depends on how quanta of energy can be accommodated within the various (e.g., translational, rotational and vibrational) microstates available. Hence, it appears that incorporation into the fibroin hydration shell limits some of the microstates available in ‘free’ water, equivalent to approximately 2R out of roughly 9R of heat capacity for free water.

While the heat capacity of ideal gases is well understood (involving only translational states), the situation for poly-atomic molecules in a condensed state is considerably more complex. In general, the heat capacity of liquids reflects the combination of (translational, rotational and vibrational) motion and intermolecular interactions [205,206,207,208,209,210,211,212]. Moreover, the description for water is further complicated due to its extensive hydrogen bonding [213,214,215,216,217,218,219,220,221,222]. A number of different (and not entirely consistent) explanations for its ‘anomalously’ high heat capacity have been proposed, based on contributions due to water clusters [213,214,215,219,220]; the distribution of rotational and vibrational energy levels [216,217,218]; temperature-driven breaking of H-bonds [219] and vibrations in the H-bonded network [220]. In the absence of a clear explanation applicable to ‘free’ water, however, a precise explanation for the decrease in heat capacity through interaction with fibroin (or other solutes) is somewhat speculative. Further investigations into these possible explanations may include measuring high frequency dielectric responses, vibrational studies in the terahertz (THz) range, inelastic neutron scattering and nuclear relaxation rates by NMR.

DSC provides the most direct method for observing changes in heat capacity, where the energy required to raise the temperature of a material (or released on lowering the temperature) is measured continuously [223,224,225]. This technique is commonly used to observe energy intake or output associated with phase transitions (e.g., peaks due to melting or protein denaturing), while changes in heat capacity appear as curvature in the baseline (e.g., ‘steps’ associated with polymer glass transitions).

It is generally recognised that thermal denaturation causes proteins to gel [32,33,34,35,36,37,38,39]. Consequently, lower levels of vibrational activity of the gelled protein would be expected, corresponding to its heat capacity decreasing. On the contrary, DSC shows that denaturation produces an increase in heat capacity [224,225,226,227,228,229,230]. The conventional explanation invokes the ‘iceberg’ model, with water becoming structured around the newly exposed hydrophobic groups, resulting in higher heat capacity. Clearly, this explanation would not fit NSF gelation, however, as our work suggests that fibroin in solution is already extensively hydrated. Instead, our model provides an alternative explanation, based on an increase in heat capacity of the water released from the hydration shell. Since this is likely to dominate any decrease in heat capacity due to the protein itself, there should be a net increase in the heat capacity. Consequently, further DSC studies to investigate changes in *C_p_* associated with NSF gelation—particularly comparing fresh and re-heated samples—could be insightful.

Using the value obtained by fitting our model (Δ*Cp* = 17.8 J K^−^^1^ mol^−^^1^ of water), the release of two water molecules per amino acid residue from the hydration shell would correspond to an increase in the heat capacity of 35.6 J K^−^^1^ mol^−^^1^ (of amino acid residues). This is somewhat smaller than the increases in heat capacity reported for denaturing various globular proteins, which range from about 41 to 76 J K^−^^1^ mol^−^^1^ (of amino acid residues) [224,225,226,227,228,229,230]. This difference may be explained, however, if the hydration water bound to a globular protein (i.e., with more restricted chain dynamics than silk fibroin) experiences a larger decrease in heat capacity. Hence, while it may not be the only factor, it appears that the release of hydration water may explain much of what has been observed for protein denaturing.

It should be emphasised that the effects discussed here result from the strong H-bonding interactions between the protein and its hydration shell. Consequently, they are unlikely to be restricted to silk fibroin, but are expected to occur more generally. In this respect, flow-induced fibril formation has recently been observed in various other proteins and peptides [231], with obvious implications for various prion diseases. Indeed, similar effects may also occur with a wider range of water-soluble polymers, such as pNiPAm [232,233].

Moreover, the ideas underlying our hypothesis regarding the hydration shell are not new, but have appeared chimera-like within the literature. For example, many studies have demonstrated slower dynamics in hydration shells around proteins, peptides and other polymers [234,235,236,237,238,239,240,241,242,243,244,245,246,247,248,249,250,251]. Furthermore, a quarter of a century ago, Dunitz [189] considered the importance of the various modes of molecular motion in solvation complexes. Nevertheless, hydration water has been overlooked, as an ‘innocent bystander’, whereas our work suggests that it should be investigated more closely as the ‘prime suspect’ in protein denaturing. This could include experimental measurements of water dynamics, using methods such as dielectric spectroscopy, NMR or inelastic neutron scattering, in addition to computational methods such as molecular dynamics simulations. Our continuing investigations in this area will be reported at a later date.

## 4. Materials and Methods

### 4.1. Materials

Fresh specimens of NSF were dissected from the middle-posterior (MP) silk gland sections of commercially reared 5th instar B. mori silkworms (four-way poly-hybrid cross of two Japanese and two Chinese strains) that were in the initial stages of cocoon construction, as described previously [9,65,99]. The protein concentrations (23 ± 2% of mainly fibroin) were determined gravimetrically, by drying NSF specimens to constant weight, in an oven at 60 °C under vacuum, on tared pieces of aluminium foil.

After peeling the membrane off the gland section (under a stereomicroscope, using fine tweezers), the specimen was transferred to the relevant apparatus, excess water was removed (by wicking away the liquid using the edge of a small piece of tissue paper) and the NSF specimen was used immediately. (It was also found that intact glands could be stored in contact with the silkworm hæmolymph for at least an hour at room temperature within a closed Petri dish, and still used without any obvious problems. Thus, in some cases, it was possible to perform two experiments with one silkworm.)

Where a concentrated protein solution was required (i.e., for IR spectroscopy), the NSF specimen was used without further treatment. Where dilute (between 0.5 and 2% *w*/*w*) protein solutions were required (i.e., for SAXS, DLS and turbidity measurements), a portion of NSF was weighed into a tared 4 mL plastic sample vial, which was subsequently topped up with type I (distilled and de-ionised) water. The sample vial was placed in a refrigerator (ca. 4 °C) and gently agitated intermittently (the vial was manually inverted a few times); the protein generally dissolved to give a clear solution within 24 h.

Where a protein film was required, a portion of NSF was placed in a polystyrene weighing boat, water (ca. 2 mL) was added, the sample was covered loosely with tissue paper and allowed to stand at ambient temperature in the laboratory. The NSF initially dissolved in the water, then a transparent protein film formed as the water evaporated. Drying was completed in an oven at 60°C under vacuum.

N-methylacetamide (NMAc, >99% purity), poly(N-isopropylacrylamide) (pNiPAm, *Mn* = 40 kDa), sodium and potassium chlorides (both >99.5% pure) were commercial materials (Sigma Aldrich, Gillingham, UK), which were used without further treatment.

### 4.2. Small-Angle X-ray Scattering (SAXS)

The SAXS pattern was collected from a portion of diluted NSF (ca. 1.2% *w*/*w*) at ambient temperature (ca. 25 to 30 °C), held within a circular liquid cell (path length ca. 2 mm, aperture 10 mm diam.) fitted with mica windows (each 25 µm thickness). The sample cell was loaded onto the sample stage of a modified Nanostar SAXS camera (Bruker, Billerica, MA, USA), fitted with a GeniX-3D generator (Xenocs, Grenoble, France) run at 50 kV and 0.6 mA, and a Hi-Star (1024 × 1024) wire grid detector. The X-ray spot (approx. 1 mm diam.) was located near the centre of the sample cell, the sample-to-detector distance was approximately 1.5 m and the scattering angular range was calibrated using a silver behenate standard. Scattering from the diluted NSF was collected in 12 successive ‘frames’, each of 300 s; the similarity in scattered intensities observed between different frames confirmed the absence of significant changes during the collection. Corrections for background intensity were made, based on scattering collected from the empty cell and after filling with water.

Changes in scattering with temperature were observed in a similar way, except that the diluted NSF solution was placed in a glass capillary (ca. 1.5 mm internal diameter), which was inserted into a Linkam heating stage. Heating rates of 0.6 or 1.0 °C min^−^^1^ were used (equivalent to a 3 or 5 °C rise for each 5 min. frame). The scattering was collected using either the modified Nanostar (as above) or a Xenocs Xeuss 2.0/Excillum camera (Xenocs, Grenoble, France, with Ga metal jet generator, Pilatus 2M detector and 2.4 m sample-to-detector distance).

### 4.3. Dynamic Light Scattering (DLS)

Diluted NSF solution was placed in a standard cuvette (10 mm path length) and loaded (at 25 °C) into a Zetasizer Nano-ZS instrument (Malvern Panalytical, Spectris, Egham, UK). Light scattering was measured at 633 nm (He-Ne laser) and a fixed scattering angle of 173°. Following a pause (120 s) to allow thermal equilibration, measurements were made in 3 blocks of 10 scans (10 s per scan). The autocorrelation function and the corresponding values of hydrodynamic radius (*R_H_*) were calculated from the data, using the instrument software, with the standard parameters for proteins in water (included in the software). The effects of heating were observed by further DLS measurements at 5 °C intervals, after allowing (120 s) thermal equilibration.

### 4.4. Infrared Spectroscopy (IR)

Infrared spectra were collected using a single beam spectrometer (Nicolet 380, Thermo-Electron Corp. Thermo-Fisher Scientific, Waltham, MA, USA) fitted with a deuterated triglycine sulphate (DTGS) detector. The optical path through the spectrometer and sample environment were purged with dry, filtered air to minimize interference due to fluctuations in atmospheric CO_2_ and water vapor. The background absorbance spectrum was collected before every experiment.

Mid range IR spectra (from 800 to 4000 cm^−^^1^) were collected in attenuated total reflectance (ATR) mode using a temperature-controlled, single bounce (45°) diamond stage (Golden Gate, SpecAc, Orpington, UK), fitted with ZnSe lenses. The specimen was placed onto the diamond internal reflectance element (IRE); solid specimens (i.e., silk protein films) were clamped in place; liquid specimens were covered with a glass coverslip to prevent evaporation. Typically, a resolution of 4 cm^−^^1^ was used; 64 scans were collected for ‘static’ specimens, while 16 or 32 scans were collected for dynamic experiments. To study spectroscopic changes with temperature, the ATR stage was programmed to heat or cool at (nominally) around 2 °C min^−^^1^; the actual temperature profile achieved (temperature vs. time) was monitored manually, using the value reported by the internal thermocouple.

Spectra in the near infrared (NIR) range (4000 to 6000 cm^−^^1^) were collected in transmission mode. Self-supporting films were held within a home-made sample holder. The same device was used to clamp liquid specimens between two glass coverslips, within an adhesive plastic ring giving a path length of around 0.25 mm. In all cases, 64 scans were collected at 4 cm^−^^1^ resolution.

### 4.5. Turbidity Measurements

An aliquot of diluted NSF (0.2 to 0.5 mL) was added to aliquot (3.0 mL) of salt solution (NaCl or KCl) of known composition, in a standard cuvette (10 mm path length), to give an initially clear solution containing around 0.1% w/w of protein. Note: the mixing could be performed at room temperature (ca. 23 °C) for solutions of NaCl below 6.5% *w*/*w* or KCl below 10% *w*/*w*; for higher concentrations, the salt solutions had to be cooled to avoid premature clouding.

Turbidity was measured (as a reduction in transmitted visible light) between 500 and 700 nm, using a home-made sample stage, heated by water circulation, in a UV/visible spectrometer (Unicam UV2, Thermo-Electron Corp. Thermo-Fisher Scientific, Waltham, MA, USA). The ‘cloud point’ was recorded as the temperature above which the turbidity increased from the initial background level.

## 5. Conclusions

We have demonstrated that fibroin in diluted solution approximates to a random coil configuration, providing capacity for extensive hydration. Thus, silk proteins appear to form a distinct subset between globular proteins with relatively dense coil geometry and IDP’s, which appear to be more expanded in aqueous solution. Comparison with other work suggests that this is also a reasonable representation of (more concentrated) fibroin in NSF.

Hydration appears to play a key role in stabilising fibroin in solution. Thermodynamic control of the hydration shell, based on favourable enthalpy but an entropic penalty, appears to be the dominant factor governing aggregation (in dilute solution) or gelation (in NSF), irrespective of protein concentration. Thus, fibroin solution is rendered thermodynamically stable under ambient conditions, but becomes unstable above 65 °C.

IR spectroscopy indicated that the hydration shell becomes less stable as the temperature is raised. Moreover, this occurred in the absence of gelation (with NMAc) or prior to gelation (in NSF), indicating that weakening of hydration is a cause (rather than consequence) of gelation.

Following observations of aggregation in dilute solution or gelation in NSF using a wide range of methods, our previous thermodynamic explanation [9] was refined. Remarkably, it was possible to express the stability of the hydration shell using a relatively simple, three parameter model. Fitting this to the data suggested that the key parameter was the reduction in heat capacity of the hydration water, which can be ascribed to its reduced vibrational or translational dynamics, due to strong H-bonding with the protein. Thus, this phenomenon is unlikely to be limited to silk fibroin, and is expected to arise with other proteins and water-soluble polymers more generally, which may inspire topics for further investigation. Indeed, these findings may point to the need to re-evaluate the foundations of the conventional explanation of protein gelation.

## Data Availability

Data is available from the authors (PRL) on request, or from the ORDA website at the University of Sheffield: https://doi.org/10.15131/shef.data.18318980 (accessed on 30 November 2021).

## Sample Availability

Samples of the compounds are not available from the authors.

## References

[1] Rudall K.M., Kenchington W. (1971). Arthropod silks: The problem of fibrous protein in animal tissues. Annu. Rev. Entomol..

[2] Kaplan D., Adams W.W., Farmer B., Viney C. (1994). Silks: Biology, structure, properties and genetics. ACS Symp. Ser..

[3] Craig C.L. (1997). Evolution of arthropod silks. Annu. Rev. Entomol..

[4] Sutherland T.D., Young J.H., Weisman S., Hayashi C.Y., Merritt D.J. (2010). Insect silk: One name, many materials. Annu. Rev. Entomol..

[5] Eisoldt L., Smith A., Scheibel T. (2011). Decoding the secrets of spider silk. Mater. Today.

[6] Walker A.A., Holland C., Sutherland T.D. (2015). More than one way to spin a crystallite: Multiple trajectories through liquid crystallinity to solid silk. Proc. R. Soc. B.

[7] Andersson M., Johansson J., Rising A. (2016). Silk spinning in spiders and silkworms. Int. J. Mol. Sci..

[8] Liu X., Zhang K.-Q., Lesieur C. (2014). Silk fiber—Molecular formation mechanism, structure-property relationship and advanced applications. Oligomerization of Chemical and Biological Compounds.

[9] Laity P.R., Holland C. (2016). The rheology behind stress-induced solidification in native silk feedstocks. Int. J. Mol. Sci..

[10] Foà C. (1912). Die kolloiden Eigenschaften der natürlichen Seide (Properties of natural silk). Zeitschr. Chem. Ind. Kolloide.

[11] Magoshi J., Magoshi Y., Becker M.A., Kato M., Han Z., Tanaka T., Inoue S.-I., Nakamura S. (2000). Crystallisation of silk fibroin from solution. Thermochim. Acta.

[12] Ochi A., Hossain K.S., Magoshi J., Nemoto N. (2002). Rheology and dynamic light scattering of silk fibroin solution extracted from the middle division of *Bombyx mori* silkworm. Biomacromolecules.

[13] Tanaka T., Magoshi J., Magoshi Y., Inoue S., Kobayashi M., Tsuda H., Becker M.A., Nakamura S. (2002). Thermal properties of *Bombyx mori* and several wild silkworm silks: Phase transition of liquid silk. J. Therm. Anal. Calorim..

[14] Nagarkar S., Nicolai T., Chassenieux C., Lele A. (2010). Structure and gelation mechanism of silk hydrogels. Phys. Chem. Chem. Phys..

[15] Moriya M., Roschzttardtz F., Nakahara Y., Saito H., Masabuchi Y., Asakura T. (2009). Rheological properties of native silk fibroins from domestic and wild silkworms, and flow analysis in each spinneret by a finite element method. Biomacromolecules.

[16] Holland C., Hawkins N., Frydrych M., Laity P., Porter D., Vollrath F. (2018). Differential scanning calorimetry of native silk feedstock. Macromol. Biosci..

[17] Chen X., Shao Z., Marinkovic N.S., Miller L.M., Zhou P., Chance M.R. (2001). Conformation transition kinetics of regenerated *Bombyx mori* silk fibroin membrane monitored by time-resolved FTIR spectroscopy. Biophys. Chem..

[18] Kim U.J., Park J., Li C., Jin H.-J., Valluzzi R., Kaplan D.L. (2004). Structure and properties of silk hydrogels. Biomacromolecules.

[19] Kasoju N., Hawkins N., Pop-Georgievski O., Kubies D., Vollrath F. (2016). Silk fibroin gelation via non-solvent induced phase separation. Biomater. Sci..

[20] Ramsden W. (1938). Coagulation by shearing and by freezing. Nature.

[21] Nguyen H.T., Luong H.T., Nguyen H.D., Tran H.A., Huynh K.C., Vo T.V. (2017). Investigate the effect of thawing process on the self-assembly of silk protein for tissue applications. BioMed Res. Int..

[22] Kolahreez D., Morshed M. (2018). Fabrication of porous three-dimensional fibroin structures through a freezing process. J. Appl. Polym. Sci..

[23] Iizuka E. (1966). Mechanism of fiber formation by the silkworm, *Bombyx mori*, L.. Biorheology.

[24] Iizuka E. (1983). The physico-chemical properties of silk fibers and the fiber spinning process. Experimentia.

[25] Iizuka E. (1985). Silk thread: Mechanism of spinning and its mechanical properties. J. Appl. Polym. Sci. Polym. Symp..

[26] Magoshi J., Magoshi Y., Nakamura S. (1985). Physical properties and structure of silk. 10: The mechanism of fibre formation from liquid silk of the silkworm *Bombyx mori*. Polym. Commun..

[27] Knight D.P., Knight M.M., Vollrath F. (2000). Beta transition and stress-induced phase separation in the spinning of spider dragline silk. Int. J. Biol. Macromol..

[28] Ohgo K., Bagusat F., Asakura T., Scheler U. (2008). Investigation of structural transition of regenerated silk fibroin aqueous solution by rheo-NMR spectroscopy. J. Am. Chem. Soc..

[29] Holland C., Urbach J.S., Blair D.L. (2012). Direct visualization of shear dependent silk fibrillogenesis. Soft Matter.

[30] Giesa T., Perry C.C., Buehler M.J. (2016). Secondary structure transition and critical stress for a model of spider silk assembly. Biomacromolecules.

[31] Sparkes J., Holland C. (2018). The energy requirements for flow-induced solidification of silk. Macromol. Biosci..

[32] Ferscht A. (1999). Structure and Mechanism in Protein Science: A Guide to Enzyme Catalysis and Protein Folding.

[33] Whitford D. (2005). Proteins: Structure and Function.

[34] Raccosta S., Manno M., Bulone D., Giacomazza D., Militello V., Martorano V., San Biagio P.L. (2010). Irreversible gelation of thermally unfolded proteins: Structural and mechanical properties of lysozyme aggregates. Eur. Biophys. J..

[35] Clark A.H., Kavanagh G.M., Ross-Murphy S.B. (2001). Globular protein gelation—theory and experiment. Food Hydrocoll..

[36] Gosal W.S., Ross-Murphy S.B. (2000). Globular protein gelation. Curr. Opin. Coll. Interface Sci..

[37] Allain A.-F., Paquin P., Subirade M. (1999). Relationships between conformation of β-lactoglobulin in solution and gel states as revealed by attenuated total reflection Fourier transform infrared spectroscopy. Int. J. Biol. Macromol..

[38] Doi E. (1993). Gels and gelling of globular proteins. Trends Food Sci. Technol..

[39] Dill K.A. (1990). Dominant forces in protein folding. Biochemistry.

[40] Wuttke R., Hofmann H., Nettels D., Borgia M.B., Mittal J., Best R.B., Schuler B. (2014). Temperature-dependent solvation modulates the dimensions of disordered proteins. Proc. Natl. Acad. Sci. USA.

[41] Tompa P. (2012). Intrinsically disordered proteins: A 10-year recap. Trends Biochem. Sci..

[42] Tompa P., Fersht A. (2009). Structure and Function of Intrinsically Disordered Proteins.

[43] Uversky V.N. (2009). Intrinsically disordered proteins and their environment: Effects of strong denaturants, temperature, pH, counter ions, membranes, binding partners, osmolytes, and macromolecular crowding. Protein J..

[44] Wright P.E., Dyson H.J. (1999). Intrinsically unstructured proteins: Re-assessing the protein structure-function paradigm. J. Mol. Biol..

[45] Iizuka E., Yang J.T. (1968). The disordered and β-conformations of silk fibroin in solution. Biochemistry.

[46] Asakura T., Suzuki H., Watanabe Y. (1983). Conformational characterization of silk fibroin in intact *Bombyx mori* and *Philosamia cynthia ricini* silkworms by ^13^C NMR spectroscopy. Macromolecules.

[47] Asakura T., Watanabe Y., Uchida A., Minagawa H. (1984). NMR of silk fibroin. 2. 13C NMR study of the chain dynamics and solution structure of *Bombyx mori* silk fibroin. Macromolecules.

[48] Asakura T., Okushita K., Williamson M.P. (2015). Analysis of the structure of *Bombyx mori* silk fibroin by NMR. Macromolecules.

[49] Hijirida D.H., Do K.G., Michal C., Wong S., Zax D., Jelinski L.W. (1996). 13C NMR of *Nephila clavipes* major ampullate silk gland. Biophys. J..

[50] Dicko C., Vollrath F., Kenney J.M. (2004). Spider silk protein refolding is controlled by changing pH. Biomacromolecules.

[51] Dicko C., Knight D., Kenney J.M., Vollrath F. (2004). Structural conformation of spidroin in solution: A synchrotron radiation circular dichroism study. Biomacromolecules.

[52] Lefèvre T., Leclerc J., Rioux-Dubé J.F., Buffeteau T., Paquin M.-C., Rousseau M.E., Cloutier I., Auger M., Gagné S.M., Boudreault S. (2007). Conformation of spider silk proteins in situ in the intact major ampullate gland and in solution. Biomacromolecules.

[53] Lefèvre T., Boudreault S., Cloutier C., Pézolet M. (2008). Conformational and orientational transformation of silk proteins in the major ampullate gland of Nephila clavipes spiders. Biomacromolecules.

[54] Xu D., Yarger J.L., Holland G.P. (2014). Exploring the backbone dynamics of native spider silk proteins in Black Widow silk glands with solution-state NMR spectroscopy. Polymer.

[55] Suzuki Y. (2016). Structures of silk fibroin before and after spinning and biomedical applications. Polym. J..

[56] Oktaviani N.A., Matsugami A., Malay A.D., Hayashi F., Kaplan D., Numata K. (2018). Conformation and dynamics of soluble repetitive domain elucidates the initial β-sheet formation of spider silk. Nat. Commun..

[57] Greving I., Terry E.A., Holland C., Boulet-Audet M., Grillo I., Vollrath F., Dicko C. (2020). Structural diversity of native major ampullate, minor ampullate, cylindriform, and flagelliform silk proteins in solution. Biomacromolecules.

[58] Asakura T., Suzuki Y., Nakazawa Y., Yazawa K., Holland G.P. (2013). Silk structure studied with nuclear magnetic resonance. Prog. Nucl. Mag. Res. Spectr..

[59] Miles A.J., Janes R.W., Wallace B.A. (2021). Tools and methods for circular dichroism spectroscopy of proteins: A tutorial review. Chem. Soc. Rev..

[60] Greving I., Dicko C., Terry A., Callow P. (2010). Vollrath, Small angle neutron scattering of native and reconstituted silk fibroin. Soft Matter.

[61] Roe R.-J. (2000). Methods of X-ray and Neutron Scattering in Polymer Science.

[62] Gedde U.W. (1999). Polymer Physics.

[63] Amino Acid Sequences Published on Genbank. https://www.ncbi.nlm.nih.gov/protein/.

[64] Ainavarapu S.R.K., Brujić J., Huang H.H., Wiita A.P., Lu H., Li L., Walther K.A., Carrion-Vazquez M., Li H., Fernandez J.M. (2007). Contour length and refolding rate of a small protein controlled by engineered disulfide bonds. Biophys. J..

[65] Laity P.R., Baldwin E., Holland C. (2018). Changes in silk feedstock rheology during cocoon construction: The role of calcium and potassium ions. Macromol. Biosci..

[66] Schaefer C., Laity P.R., Holland C., McLeish T.C.B. (2020). Silk protein solution: A natural example of sticky reputation. Macromolecules.

[67] Schaefer C., Laity P.R., Holland C., McLeish T.C.B. (2021). Stretching of Bombyx mori silk protein in flow. Molecules.

[68] Martel A., Burghammer M., Davies R., DiCola E., Panine P., Salmon J.-B., Riekel C. (2008). A microfluidic cell for studying the formation of regenerated silk by synchrotron radiation small- and wide-angle X-ray scattering. Biomicrofluidics.

[69] Hol W.G.J. (1985). Effects of the α-helix dipole upon the functioning and structure of proteins and peptides. Adv. Biophys..

[70] Sengupta D., Behera R.N., Smith J.C., Ullmann G.M. (2005). The α-helix dipole: Screened out?. Structure.

[71] Wilkins D.K., Grimshaw S.B., Receveur V., Dobson C.M., Jones J.A., Smith L.J. (1999). Hydrodynamic radii of native and denatured proteins measured by pulse field gradient NMR techniques. Biochemistry.

[72] Damaschun G., Damaschun H., Gast K., Zirwer D. (1998). Denatured states of yeast phosphoglycerate kinase. Biochemistry.

[73] Zhou H.-X. (2002). Dimensions of denatured protein chains from hydrodynamic data. J. Phys. Chem. B.

[74] Bernadó P., Blackledge M. (2009). A self-consistent description of the conformational behavior of chemically denatured proteins from NMR and small angle scattering. Biophys. J..

[75] Rawat N., Biswas P. (2009). Size, shape, and flexibility of proteins and DNA. J. Chem. Phys..

[76] Uversky V.N. (2013). The alphabet of intrinsic disorder II. Various roles of glutamic acid in ordered and intrinsically disordered proteins. Proteins.

[77] Machii H. (1989). Varietal differences of nitrogen and amino acid contents in mulbery leaves. Acta Sericol. Entomol..

[78] Machii H., Koyama A., Yamanouchi H. Mulberry breeding, cultivation and utilisation in Japan. Proceedings of the FAO Electronic Conference on Mulberry for Animal Production.

[79] Hansen J.C., Lu X., Ross E.D., Woody R.W. (2006). Intrinsic protein disorder, amino acid composition and histone terminal domains. J. Biol. Chem..

[80] He Y.-X., Zhang N.-N., Li W.-F., Jia N., Chen B.-Y., Zhou K., Zhang J., Chen Y., Zhou C.-Z. (2012). N-terminal domain of *Bombyx mori* fibroin mediates the assembly of silk in response to pH decrease. J. Mol. Biol..

[81] Eisoldt L., Thamm C., Scheibel T. (2011). Review: The role of terminal domains during storage and assembly of spider silk proteins. Biopolymers.

[82] Hagn F., Thamm C., Scheibel T., Kessler H. (2011). pH-dependent dimerization and salt-dependent stabilization of the N-terminal domain of spider dragline silk—implications for fiber formation. Angew. Chem. Int. Ed..

[83] Bauer J., Scheibel T. (2017). Dimerization of the conserved N-terminal domain of a spider silk protein controls the self-assembly of the repetitive core domain. Biomacromolecules.

[84] Kronqvist N., Otikovs M., Chmyrov V., Chen G., Andersson M., Nordling K., Landreh M., Sarr M., Jörnvall H., Wennmalm S. (2014). Sequential pH-driven dimerization and stabilization of the N-terminal domain enables rapid spider silk formation. Nat. Commun..

[85] Andersson M., Chen G., Otikovs M., Landreh M., Nordling K., Kronqvist N., Westermark P., Jörnvall H., Knight S., Ridderstråle Y. (2014). Carbonic anhydrase generates CO_2_ and H^+^ that drive spider silk formation via opposite effects on the terminal domains. PLoS Biol..

[86] Schwarze S., Zwettler F.U., Johnson C.M., Neuweiler H. (2013). The N-terminal domains of spider silk proteins assemble ultrafast and protected from charge screening. Nat. Commun..

[87] Barrosa da Silva F.L., Pasquali S., Derreumaux P., Dias L.G. (2016). Electrostatics analysis of the mutational and pH effects of the N-terminal domain self-association of the major ampullate spidroin. Soft Matter.

[88] Gauthier M., Leclerc J., Lefèvre T., Gagné S.M., Auger M. (2014). Effect of pH on the structure of the recombinant C-terminal domain of Nephila clavipes dragline silk protein. Biomacromolecules.

[89] Gaines W.A., Sehorn M.G., Marcotte W.R. (2010). Spidroin N-terminal domain promotes a pH-dependent association of silk proteins during self-assembly. J. Biol. Chem..

[90] Hossain K.S., Nemoto N., Magoshi J. (1999). Dynamic and static light scattering of dilute aqueous solutions of silk fibroin collected from Bombyx mori silkworms. Langmuir.

[91] Jin H.-J., Kaplan D.L. (2003). Mechanism of silk processing in insects and spiders. Nature.

[92] Lu S., Li J., Zhang S., Yin Z., Xing T., Kaplan D. (2015). The influence of the hydrophilic–lipophilic environment on the structure of silk fibroin protein. J. Mater. Chem. B.

[93] Malay A.D., Suzuki T., Katashima T., Kono N., Arakawa K., Numata K. (2020). Spider silk self-assembly via modular liquid-liquid phase separation and nanofibrillation. Sci. Adv..

[94] Seib F.P. (2021). Emerging silk material trends: Repurposing, phase separation and solution-based designs. Materials.

[95] Kyte J., Doolittle R.F. (1982). A simple method for displaying the hydropathic character of a protein. J. Mol. Biol..

[96] Makhatadze G.I., Privalov P.L. (1993). Contribution of hydration to protein folding thermodynamics: I the enthalpy of hydration. J. Mol. Biol..

[97] Privalov P.L., Makhatadze G.I. (1993). Contribution of hydration to protein folding thermodynamics: II the entropy and Gibbs energy of hydration. J. Mol. Biol..

[98] Laity P.R., Gilks S.E., Holland C. (2015). Rheological behaviour of native silk feedstocks. Polymer.

[99] Cowie J.M.G. (1997). Polymers: Chemistry and Physics of Modern Materials.

[100] Flippen R.B., Barth H.G., Mays J.W. (1991). Photon correlation spectroscopy. Modern Methods of Polymer Characterization.

[101] Berry G.C., Cotts P.M., Pethrick R.A., Dawkins J.V. (1999). Static and dynamic light scattering. Modern Techniques for Polymer Characterisation.

[102] Pecora R. (2000). Dynamic light scattering measurement of nanometer particles in liquids. J. Nanopart. Res..

[103] Hossain K., Ochi A., Ooyama E., Magoshi J., Nemoto N. (2003). Dynamic light scattering of native silk fibroin solution extracted from different parts of the middle division of the silk gland of the *Bombyx mori* silkworm. Biomacromolecules.

[104] Falk M., Ford T.A. (1966). Infrared spectrum and structure of liquid water. Can. J. Chem..

[105] Brubach J.-B., Mermet A., Filabozzi A., Gerschel A., Roy P. (2005). Signatures of the hydrogen bonding in the infrared bands of water. J. Chem. Phys..

[106] Auer B.M., Skinner J.L. (2008). IR and Raman spectra of liquid water: Theory and interpretation. J. Chem. Phys..

[107] Carpenter W.B., Fournier J.A., Biswas R., Voth G.A., Tokmakov A. (2017). Delocalization and stretch-bend mixing of the HOH bend in liquid water. J. Chem. Phys..

[108] Kananenka A.A., Skinner J.L. (2018). Fermi resonance in OH-stretch vibrational spectroscopy of liquid water and the water hexamer. J. Chem. Phys..

[109] Hunter K.M., Shakib F.A., Paesani F. (2018). Disentangling coupling effects in the infrared spectra of liquid water. J. Phys. Chem. B.

[110] Grechko M., Hasegawa T., D’Angelo F., Ito H., Turchinovich D., Nagata Y., Bonn M. (2018). Coupling between intra- and intermolecular motions in liquid water revealed by two dimensional terahertz-infrared-visible spectroscopy. Nat. Commun..

[111] Zhang B., Yu Y., Zhang Y.-Y., Jiang S., Li Q., Hu H.-S., Li G., Zhao Z., Wang C., Xie H. (2020). Infrared spectroscopy of neutral water clusters at finite temperature: Evidence for a noncyclic pentamer. Proc. Natl. Acad. Sci. USA.

[112] Barth A., Zscherp C. (2020). What vibrations tell us about proteins. Quart. Rev. Biophys..

[113] Barth A. (2007). Infrared spectroscopy of proteins. Biochim. Biophys. Acta.

[114] Fabian H., Mäntele W. (2006). Infrared spectroscopies of proteins. Handbook of Vibrational Spectroscopy.

[115] Harrick N.J. (1960). Surface chemistry from spectral analysis of totally internally reflected radiation. J. Phys. Chem..

[116] Ekgasit S., Padermshoke A. (2001). Optical contact in ATR/FT-IR spectroscopy. Appl. Spectr..

[117] Milosevic M. (2004). Internal reflection and ATR spectroscopy. Appl. Spectr. Rev..

[118] Boulet-Audet M., Buffeteau T., Boudreault S., Daugey N., Pézolet M. (2010). Quantitative determination of band distortions in diamond attenuated total reflectance infrared spectra. J. Phys. Chem. B..

[119] Corujo M.P., Sklepari M., And D.L., Millichip M., Reason A., Goodchild S.C., Wormell P., Amarasinghe D.P., Lindo V., Chmel N.P. (2018). Infrared absorbance spectroscopy of aqueous proteins: Comparison of transmission and ATR data collection and analysis of structure fitting. Chirality.

[120] Chen X.G., Schweitzer-Stenner R., Krimm S., Mirkin N.G., Asher S.A. (1994). N-methylacetamide and its hydrogen bonded water molecules are vibrationally coupled. J. Am. Chem. Soc..

[121] Cazade P.-A., Hédin F., Xu Z.-H., Meuwly M. (2015). Vibrational relaxation and energy migration of N-methylacetamide in water: The role of nonbonded interactions. J. Phys. Chem. B..

[122] Panuszko A., Gojlo E., Zielkiewicz J., Śmiechowski M., Krakowiak J., Stangret J. (2008). Hydration of simple amides. FTIR spectra of HDO and theoretical studies. J. Phys. Chem. B..

[123] Panuszko A., Wojciechowski M., Bruździak P., Rakowska P.W., Stangrat J. (2012). Characteristics of hydration water around hen egg lysozyme as the protein model in aqueous solution. FTIR spectroscopy and molecular dynamics simulation. Phys. Chem. Chem. Phys..

[124] Panuszko A., Nowak M.G., Bruździak P., Stasiulewicz M., Stangrat J. (2019). Amides as models to study the hydration of proteins and peptides—Spectroscopic and theoretical approach on hydration in various temperatures. J. Mol. Liq..

[125] Iwamoto R. (2010). Infrared and near-infrared study of the interaction of amide C=O with water in ideally inert medium. J. Phys. Chem. A.

[126] Allison S.K., Bates S.P., Crain J., Martyna G.J. (2006). Solution structure of the aqueous model peptide N-methylacetamide. J. Phys. Chem. B.

[127] Tan J., Zhang J., Li C., Luo Y., Ye S. (2019). Ultrafast energy relaxation dynamics of amide I vibrations coupled with protein-bound water molecules. Nat. Commun..

[128] Dannenberg J.J. (2006). Enthalpies of hydration of N-methylacetamide by one, two, and three waters and the effect upon the C=O stretching frequency, an ab initio DFT study. J. Phys. Chem. A.

[129] Dixon D.A., Dobbs K.D., Valentini J.J. (1994). Amide-water and amide-amide hydrogen bond strengths. J. Phys. Chem..

[130] Xiao X., Tan Y., Zhu L., Guo Y., Wen Z., Li M., Pu X., Tian A. (2012). Effects of the position and manner of hydration on the stability of solvated N-methylacetamides and the strength of binding between N-methylacetamide and water clusters: A computational study. J. Mol. Model..

[131] Cai K., Su T., Lin S., Zheng R. (2014). Molecular mechanics force field-based general map for the solvation effect on amide I probe of peptide in different micro-environments. Spectrochim. Acta A Mol. Biomol. Spectr..

[132] Yadav V.K., Chandra A. (2015). First-principles simulation study of vibrational spectral diffusion and hydrogen bond fluctuations in aqueous solution of N-methylacetamide. J. Phys. Chem. B..

[133] Amunson K., Kubelka J. (2007). On the temperature dependence of amide I frequencies of peptides in solution. J. Phys. Chem. B..

[134] Ingrosso F., Monard G., Farag M.H., Bastida A., Ruiz-López M.F. (2011). Importance of polarisation and charge transfer effects to model the infrared spectra of peptides in solution. J. Chem. Theory Comput..

[135] Donati G., Petrone A., Rega N. (2020). Multiresolution continuous wavelet transform for studying coupled solute–solvent vibrations via ab initio molecular dynamics. Phys. Chem. Chem. Phys..

[136] Jorgensen W.L., Swenson C.J. (1985). Optimized intermolecular potential functions for amides and peptides. Hydration of amides. J. Am. Chem. Soc..

[137] Jorgensen W.L., Gao J. (1988). Cis-trans energy difference for the peptide bond in the gas phase and aqueous solution. J. Am. Chem. Soc..

[138] Gaigeot M.P., Vuilleumier R., Sprik M., Borgis D. (2005). Infrared spectroscopy of N-methylacetamide revisited by ab initio molecular dynamics simulations. J. Chem. Theory Comput..

[139] Deetz M.J., Fahey J.E., Smith B.D. (2001). NMR studies of hydrogen bonding interactions with secondary amide and urea groups. J. Phys. Org. Chem..

[140] Di Gioacchino M., Bruni F., Ricci M.A. (2019). N-methylacetamide aqueous solutions: A neutron diffraction study. J. Phys. Chem. B.

[141] Kang Y.K., Park H.S. (2004). Internal rotation about the C-N bond of amides. J. Mol. Struct..

[142] Mantz Y.A., Branduardi D., Bussi G., Parrinello M. (2009). Ensemble of transition state structures for the cis-trans isomerization of N-methylacetamide. J. Phys. Chem. B..

[143] Stark E., Luchter K., Margoshes M. (1986). Near-infrared analysis (NIRA): A technology for quantitative and qualitative analysis. Appl. Spectr. Revs..

[144] Bokobza L. (1998). Near infrared spectroscopy. J. Near Infrared Anal..

[145] Pasquini C. (2003). Near infrared spectroscopy: Fundamentals, practical aspects and analytical applications. J. Braz. Chem. Soc..

[146] Burns D.A., Ciurczak E.W. (2008). Handbook of Near-Infrared Analysis.

[147] Vandermuelen D.L., Ressler N. (1980). A near-infrared analysis of water-macromolecule interactions: Hydration and the spectra of aqueous solutions of intact proteins. Arch. Biochem. Biophys..

[148] Lamanna R., Delmelle M., Cannistraro S. (1990). A near-infrared study of hydrogen bonding in human albumin aqueous solutions. Chem. Phys. Lett..

[149] Liu Y., Czarnecki M.A., Ozaki Y. (1993). Fourier-transform near-infrared study of dissociation and thermodynamic properties of N-methylacetamide in a carbon tetrachloride solution. Bull. Inst. Chem. Res. Kyoto Univ..

[150] Wang J., Sowa M.G., Ahmed K., Mantsch H.H. (1994). Photoacoustic near-infrared investigation of homo-polypeptides. J. Phys. Chem..

[151] Czarnecki M.A., Haufa K.Z. (2005). Effect of temperature and concentration on the structure of N-methylacetamide-water complexes: Near-infrared spectroscopic study. J. Phys. Chem. A..

[152] Izutsu K., Fujimaki Y., Kuwabara A., Hiyama Y., Yomota C., Aoyagi N. (2006). Near-infrared analysis of protein secondary structure in aqueous solutions and freeze-dried solids. J. Pharm. Sci..

[153] Ma L., Cui X., Cai W., Shao X. (2018). Understanding the function of water during the gelation of globular proteins by temperature dependent near infrared spectroscopy. Phys. Chem. Chem. Phys..

[154] Mo C., Wu P., Chen X., Shao Z. (2006). Near-infrared characterization on the secondary structure of regenerated Bombyx mori silk fibroin. Appl. Spectr..

[155] Mo C., Wu P., Chen X., Shao Z. (2009). The effect of water on the conformation transition of Bombyx mori silk fibroin. Vibr. Spectr..

[156] Mapelli M., Greco F., Gussoni M., Consonni R., Zetta L. (1997). Application of NMR microscopy to the morphological study of the silkworm Bombyx mori during its metamorphosis. Mag. Res. Imag..

[157] Baldwin R.L. (1996). How Hofmeister ion interactions affect protein stability. Biophys. J..

[158] Zhang Y., Cremer P.S. (2006). Interactions between macromolecules and ions: The Hofmeister series. Curr. Opin. Chem. Biol..

[159] Sedlák E., Stagg L., Wittung-Stafshede P. (2008). Effect of Hofmeister ions on protein thermal stability: Roles of ionhydration and peptide groups?. Arch. Biochem. Biophys..

[160] Salis A., Ninham B.W. (2014). Models and mechanisms of Hofmeister effects in electrolyte solutions, and colloid and protein systems revisited. Chem. Soc. Rev..

[161] Senske M., Constantinescu-Aruxandei D., Havenith M., Herrmann C., Weingärtner H., Ebbinghaus S. (2016). The temperature dependence of the Hofmeister series: Thermodynamic fingerprints of cosolute–protein interactions. Phys. Chem. Chem. Phys..

[162] Debye P. (1944). Light scattering in solutions. J. Appl. Phys..

[163] Doty P., Steiner R.F. (1950). Light scattering and spectrophotometry of colloidal solutions. J. Chem. Phys..

[164] Parker T.G., Dalgleish D.G. (1977). The use of light scattering and turbidity measurements to study the kinetics of extensively aggregated proteins: αs-casein. Biopolymers.

[165] Camerini-Otero R.D., Day L.A. (1978). The wavelength dependence of the turbidity of solutions of macromolecules. Biopolymers.

[166] Hall D., Minton A.P. (2005). Turbidity as a probe of tubulin polymerisation kinetics: A theoretical and experimental re-examination. Anal. Biochem..

[167] Grollman A. (1931). The vapour pressure of aqueous solutions with special reference to the problem of the state of water in biological fluids. J. Gen. Physiol..

[168] Robinson R.A. (1945). The vapour pressure of solutions of potassium chloride and sodium chloride. Trans. R. Soc. N. Z..

[169] Robinson R.A. (1961). Activity coefficients of sodium chloride and potassium chloride in mixed aqueous solutions at 25°. J. Phys. Chem..

[170] Robinson R.A., Bower V.E. (1965). An additivity rule for the vapour pressure lowering of aqueous solutions. J. Res. Nat. Bureau Stand. A Phys. Chem..

[171] Sadeghi R., Ziamajidi F. (2006). Water activities of ternary mixtures of poly(ethylene glycol), NaCl and water over the temperature range of 293.15 K to 313.15 K. J. Chem. Thermodyn..

[172] Wagner W., Pruß A. (2002). The IAPWS formulation 1995 for the thermodynamic properties of ordinary water substance for general and scientific use. J. Phys. Chem. Ref. Data.

[173] Feistel R., Wagner W. (2006). A new equation of state for H_2_O ice Ih. J. Phys. Chem. Ref. Data.

[174] Reiss H., Wilson I.B. (1948). The effect of surface on melting point. J. Colloid Sci..

[175] Still R.C., Skapski A.S. (1956). Method for the determination of the surface tension of solids from their melting points in thin wedges. J. Chem. Phys..

[176] Hay J.N., Laity P.R. (2000). Observations of water migration during thermoporometry studies of cellulose films. Polymer.

[177] Laity P.R., Holland C. (2017). Thermo-rheological behaviour of native silk feedstocks. Eur. Polym. J..

[178] Russell T.P., Hjelm R.P., Seeger P.A. (1990). Temperature dependence of the interaction parameter of polystyrene and poly(methyl methacrylate). Macromolecules.

[179] Etxabarren C., Iriarte M., Uriarte C., Etxeberrie A., Iruin J.J. (2002). Polymer-solvent interaction parameter in polymer solutions at high polymer concentrations. J. Chromatog..

[180] Dudowicz J., Freed K.F., Douglas J.F. (2013). Solvation of polymers as mutual association. I. General theory. J. Chem. Phys..

[181] Dudowicz J., Freed K.F., Douglas J.F. (2013). Solvation of polymers as mutual association. II. Basic thermodynamic properties. J. Chem. Phys..

[182] Mills E.A., Plotkin S.S. (2015). Protein transfer free energy obeys entropy-enthalpy compensation. J. Phys. Chem. B.

[183] Pan A., Kar T., Rakshit A., Moulik S.P. (2016). Enthalpy–entropy compensation (eec) effect: Decisive role of free energy. J. Phys. Chem. B..

[184] Bigman L.S., Levy Y. (2018). Entropy-enthalpy compensation in conjugated proteins. Chem. Phys..

[185] Khrapunov S. (2018). The enthalpy-entropy compensation phenomenon. Limitations for the use of some basic thermodynamic equations. Curr. Protein Pept. Sci..

[186] Moulik S.P., Naskar B., Rakshit A.K. (2019). Current status of enthalpy-entropy compensation phenomenon. Curr. Sci..

[187] Wang X., Yang X., Chen H., Yang X., Xu Z. (2020). Entropy–enthalpy compensation in peptide adsorption on solid surfaces: Dependence on surface hydration. Langmuir.

[188] Dunitz J.D. (1995). Win some, lose some: Enthalpy-entropy compensation in weak intermolecular interactions. Chem. Biol..

[189] Hu X., Kaplan D., Cebe P. (2008). Dynamic protein-water relationships during β-sheet formation. Macromolecules.

[190] Michnik A. (2003). Thermal stability of bovine serum albumin: DSC study. J. Thermal Anal. Calorim..

[191] Michnik A., Michalik K., Kluczewska A., Drzazga Z. (2006). Comparative dsc study of human and bovine serum albumin. J. Therm. Anal. Calorim..

[192] Weijers M., Barneveld P.A., Cohen-Stuart M.A., Visschers R.W. (2003). Heat-induced denaturation and aggregation of ovalbumin at neutral pH described by irreversible first-order kinetics. Protein Sci..

[193] Takahashi Y., Gehoh M., Yazuriha K. (1991). Crystal structure of silk (*Bombyx mori*). J. Polym. Sci. B Polym. Phys..

[194] Marsh R.E., Corey R.B., Pauling L. (1955). An investigation of the structure of silk fibroin. Biochim. Biophys. Acta.

[195] Warwicker J.O. (1954). The crystal structure of silk fibroin. Acta Cryst..

[196] Öjelund G., Sköld R., Wadsö I. (1976). Thermochemistry of solutions of biochemical model compounds, 5. Transfer of N-alkylamides from water to non-aqueous media. J. Chem. Thermodyn..

[197] Della Gatta G., Barone G., Elia V. (1986). Enthalpies of solvation for N-alkylamides in water and carbon tetrachloride at 25 °C. J. Solut. Chem..

[198] Spencer J.N., Berger S.K., Powell C.R., Henning B.D., Furman G.S., Loffredo W.M., Rydberg E.M., Neubert R.A., Shoop C.E., Blauch D.N. (1981). Amide interactions in aqueous and organic medium. J. Phys. Chem..

[199] Akiyama M. (2002). Study on hydration enthalpy of N-methylacetamide in water. Spectrochim. Acta A.

[200] Kreis R.W., Wood R.H. (1969). Enthalpy of fusion and cryoscopic constant of N-methylacetamide. J. Chem. Thermodyn..

[201] Ahlers J., Lohmann J., Gmehling J. (1999). Binary solid-liquid equilibria of organic systems containing different amides and sulfolane. J. Chem. Eng. Data.

[202] Specific Heat of Some Liquids and Fluids, The Engineering Toolbox. https://www.engineeringtoolbox.com/specific-heat-fluids-d_151.html.

[203] Lide D.R. (2001). Handbook of Chemistry and Physics.

[204] Bondi A. (1966). Estimation of the heat capacity of liquids. Ind. Eng. Chem. Fundam..

[205] Shaw R. (1969). Heat capacities of liquids. Estimation of heat capacity at constant pressure and 25 °C, using additivity rules. J. Chem. Eng. Data.

[206] Cerdeiriña C.A., Gonzáles-Salgado D., Romani L., del Carmen Dalgado M., Torres L.A., Costas M. (2004). Towards an understanding of the heat capacity of liquids. A simple two-state model for molecular association. J. Chem. Phys..

[207] Al-Shorachi H.N., Hashim E.T. (2007). Estimation of heat capacity for liquids. Pet. Sci. Technol..

[208] Bolmatov D., Brazhkin V.V., Trachenko K. (2012). The phonon theory of liquid thermodynamics. Sci. Rep..

[209] Schliesser J.M., Woodfield B.F. (2015). Development of a Debye heat capacity model for vibrational modes with a gap in the density of states. J. Phys. Condens. Matter.

[210] Naef R. (2019). Calculations of the isobaric heat capacities of the liquid and solid phase of organic compounds at and around 298.15 K based on their ‘true’ molecular volume. Molecules.

[211] Fomin Y.D. (2019). Anomalously high heat capacity of liquids, relation to structural properties. Mol. Phys..

[212] Benson S.W. (1978). Heat capacity and structure in liquids. Application to the structure of water. J. Am. Chem. Soc..

[213] Benson S.W., Siebert E.D. (1992). A simple two-structure model for liquid water. J. Am. Chem. Soc..

[214] Sceats M.G., Rice S.A. (1980). The enthalpy and heat capacity of liquid water and the ice polymorphs from a random network model. J. Chem Phys..

[215] Vidler M., Tennyson J. (2000). Accurate partition function and thermodynamic data for water. J. Chem. Phys..

[216] Abe H. (2004). Estimation of heat capacity and properties of water by spectrum decomposition of the second overtone band of OH stretching vibration. J. Near Infrared Spectrosc..

[217] Lishchuk S.V., Malomuzh N.P., Makhlaichuk P.V. (2011). Contribution of H-bond vibrations to heat capacity of water. Phys. Lett. A.

[218] Makhlaichuk V.N., Malomuzh N.P. (2018). Manifestation of cluster excitations in dielectric properties of water vapor and liquid water as well as their heat capacity. J. Mol. Liq..

[219] Chen H., Hansen K. (2014). Low temperature heat capacity of water clusters. Chem. Phys. Lett..

[220] Putintsev N.M., Putintsev D.N. (2017). Heat capacity and thermal expansion of water and helium. J. Therm. Sci..

[221] Marshall B.D. (2019). A resummed thermodynamic perturbation theory for positive and negative hydrogen bond cooperativity in water. J. Phys. Condens. Matter.

[222] Höhne G.W.H., Hemminger W.F., Flammersheim H.-J. (2003). Differential Scanning Calorimetry.

[223] Spink C.H. (2008). Differential scanning calorimetry. Methods Cell Biol..

[224] Privalov P.L. (2015). Microcalorimetry of macromolecules: The physical basis of biological structures. J. Solut. Chem..

[225] Sochava I.V., Smirnova O.I. (1993). Heat capacity of hydrated and dehydrated globular proteins: Denaturation increment of heat capacity. Food Hydrocoll..

[226] Myers J.K., Pace C.N., Scholtz J.M. (1995). Denaturant m values and heat capacity changes: Relation to changes in accessible surface areas of protein unfolding. Protein Sci..

[227] Makhatadze G.I. (1998). Heat capacities of amino acids, peptides and proteins. Biophys. Chem..

[228] Mazurenko S., Kunka A., Beerens K., Johnson C.M., Damborsky J., Prokop Z. (2017). Exploration of protein unfolding by modelling calorimetry data from reheating. Sci. Rep..

[229] Li-Blatter X., Seelig J. (2019). Thermal and chemical unfolding of lysozyme. multistate Zimm−Bragg theory versus two-state model. J. Phys. Chem. B.

[230] Shen Y., Ruggeri F.S., Vigolo D., Kamada A., Qamar S., Levin A., Iserman C., Albert S., St. George-Hyslop P., Knowles T.P.J. (2020). Biomolecular condensates undergo a generic shear-mediated liquid-to-solid transition. Nat. Nanotech..

[231] Grinberg V.Y., Burova T.V., Grinberg N.V., Moskalets A.P., Dubovik A.S., Plashchina I.G., Khokhlov A.R. (2020). Energetics and mechanisms of poly(n-isopropylacrylamide) phase transitions in water−methanol solutions. Macromolecules.

[232] Kametani S., Sekine S., Ohkubo T., Hirano T., Ute K., Cheng H.N., Asakura T. (2017). NMR studies of water dynamics during sol-to-gel transition of poly(N-isopropylacrylamide) in concentrated aqueous solution. Polymer.

[233] Halle B. (2004). Protein hydration dynamics in solution: A critical survey. Philos. Trans. Biol. Sci..

[234] Ebbinghaus S., Kim S.J., Heyden M., Yu X., Heugen U., Gruebele M., Leitner D.M., Havenith M. (2007). An extended dynamical hydration shell around proteins. Proc. Natl. Acad. Sci. USA.

[235] Ebbinghaus S., Kim S.J., Heyden M., Yu X., Heugen U., Gruebele M., Leitner D.M., Havenith M. (2008). Protein sequence and pH-dependent hydration probed by Terahertz spectroscopy. J. Am. Chem. Soc..

[236] Russo D., Ollivier J., Teixera J. (2008). Water hydrogen bond analysis on hydrophilic and hydrophobic biomolecule sites. Phys. Chem. Chem. Phys..

[237] Grebenkov D.S., Goddard Y.A., Diakova G., Korb J.-P., Bryant R.G. (2009). Dimensionality of diffusive exploration at the protein interface in solution. J. Phys. Chem. B..

[238] Perticaroli S., Comez L., Paolantoni M., Sassi P., Morresi A., Fioretto D. (2011). Extended frequency range depolarised light scattering study of N-acetyl-leucine-methylamide–water solutions. J. Am. Chem. Soc..

[239] Gallat F.-X., Laganowsky A., Wood K., Gabel F., van Eijck L., Wuttke J., Moulin M., Härtlein M., Eisenberg D., Colletier J.-P. (2012). Dynamical coupling of intrinsically disordered proteins and their hydration water: Comparison with folded soluble and membrane proteins. Biophys. J..

[240] King J.T., Kubarych K.J. (2012). Site-specific coupling of hydration water and protein flexibility studied in solution with ultrafast 2D-IR spectroscopy. J. Am. Chem. Soc..

[241] Nickels J.D., O’Neill H., Hong L., Tyagi M., Ehlers G., Weiss K.L., Zhang Q., Yi Z., Mamontov E., Smith J.C. (2012). Dynamics of protein and its hydration water: Neutron scattering studies in fully deuterated GFP. Biophys. J..

[242] Conti-Nibali V., Havenith M. (2014). New insights into the role of water in biological function: Studying solvated biomolecules using terahertz absorption spectroscopy in conjunction with molecular dynamics simulations. J. Am. Chem. Soc..

[243] Xu Y., Havenith M. (2015). Perspective: Watching low-frequency vibrations of water in biomolecular recognition by THz spectroscopy. J. Chem. Phys..

[244] Jose J.C., Khatua P., Bansal N., Sengupta N., Bandyopadhyay S. (2014). Microscopic hydration properties of the Aβ_1-42_ peptide monomer and the globular protein ubiquitin: A comparative molecular dynamics study. J. Phys. Chem. B..

[245] Sushko O., Dubrovka R., Donnan R.S. (2015). Sub-terahertz spectroscopy reveals that proteins influence the properties of water at greater distance than previously detected. J. Chem. Phys..

[246] Bellissent-Funel M.-C., Hassanali A., Havenith M., Henchman R., Pohl P., Sterpone F., van der Spoel D., Yu Y., Garcia A.E. (2016). Water determines the structure and dynamics of proteins. Chem. Rev..

[247] Serin G., Nguyen H.H., Marty J.-D., Micheau J.-C., Gernigon V., Mingotaud A.-F., Bajon D., Soulet T., Massenot S., Coudret C. (2016). Terahertz time-domain spectroscopy of thermoresponsive polymers in aqueous solution. J. Phys. Chem. B.

[248] Braun D., Schmollngruber M., Steinhauser O. (2016). Rotational dynamics of water molecules near biological surfaces with implications for nuclear quadrupole relaxation. Phys. Chem. Chem. Phys..

[249] Gavrilov Y., Leuchter J.D., Levy J. (2017). On the coupling between the dynamics of protein and water. Phys. Chem. Chem. Phys..

[250] Yamamoto N., Ito S., Nakanishi M., Chatani E., Inoue K., Kandori H., Tominaga K. (2018). Effect of temperature and hydration level on purple membrane dynamics studied using broadband dielectric spectroscopy from sub-GHz to THz regions. J. Phys. Chem. B.

[251] Batys P., Zhang Y., Lutkenhaus J.L., Sammalkorpi M. (2018). Hydration and temperature response of water mobility in poly(diallyldimethylammonium)–poly(sodium 4-styrenesulfonate) complexes. Macromolecules.

